# Psychiatric disorders converge on common pathways but diverge in cellular context, spatial distribution, and directionality of genetic effects

**DOI:** 10.1101/2025.07.11.25331381

**Published:** 2025-07-16

**Authors:** Worrawat Engchuan, Omar Shanta, Kuldeep Kumar, Jeffrey R. MacDonald, Bhooma Thiruvahindrapuram, Omar Hamdan, Marieke Klein, Adam Maihofer, James Guevara, Oanh Hong, Guillaume Huguet, Molly Sacks, Mohammad Ahangari, Rayssa M.M.W. Feitosa, Kara Han, Marla Mendes, Xiaopu Zhou, Nelson X. Bautista, Giovanna Pellecchia, Zhouzhi Wang, Daniele Merico, Ryan K.C. Yuen, Brett Trost, Ida Sønderby, Mark J. Adams, Rolf Adolfsson, Ingrid Agartz, Allison E. Aiello, Martin Alda, Judith Allardyce, Ananda B. Amstadter, Till F.M. Andlauer, Ole A. Andreassen, María S. Artigas, S. Bryn Austin, Muhammad Ayub, Dewleen G. Baker, Nick Bass, Bernhard T. Baune, Maximilian Bayas, Klaus Berger, Joanna M. Biernacka, Tim Bigdeli, Jonathan I. Bisson, Douglas Blackwood, Marco Boks, David Braff, Elvira Bramon, Gerome Breen, Tanja Brueckl, Richard A. Bryant, Cynthia M. Bulik, Joseph Buxbaum, Murray J. Cairns, Jose M. Caldas-de-Almeida, Megan Campbell, Dominique Campion, Vaughan J. Carr, Enrique Castelao, Boris Chaumette, Sven Cichon, David Cohen, Aiden Corvin, Nicholas Craddock, Jennifer Crosbie, Darrina Czamara, Udo Dannlowski, Franziska Degenhardt, Douglas L. Delahanty, Astrid Dempfle, Guillaume Desachy, Arianna Di Florio, Faith B. Dickerson, Srdjan Djurovic, Katharina Domschke, Lisa Douglas, Ole K. Drange, Laramie E. Duncan, Howard J. Edenberg, Tonu Esko, Steve Faraone, Norah C. Feeny, Andreas J. Forstner, Barbara Franke, Mark Frye, Dong-jing Fu, Janice M. Fullerton, Anna Gareeva, Linda Garvert, Justine M. Gatt, Pablo Gejman, Daniel H. Geschwind, Ina Giegling, Stephen J. Glatt, Joe Glessner, Fernando S. Goes, Katherine Gordon-Smith, Hans Grabe, Melissa J. Green, Michael F. Green, Tiffany Greenwood, Maria Grigoroiu-Serbanescu, Raquel E. Gur, Ruben C. Gur, Jose Guzman-Parra, Jan Haavik, Tim Hahn, Hakon Hakonarson, Joachim Hallmayer, Marian L. Hamshere, Annette M. Hartmann, Arsalan Hassan, Caroline Hayward, Johannes Hebebrand, Sian M.J. Hemmings, Stefan Herms, Marisol Herrera-Rivero, Anke Hinney, Georg Homuth, Andrés Ingason, Lucas T. Ito, Nakao Iwata, Ian Jones, Lisa A. Jones, Lina Jonsson, Erik G. Jönsson, René S. Kahn, Robert Karlsson, Milissa L. Kaufman, John R. Kelsoe, James L. Kennedy, Anthony King, Tilo Kircher, George Kirov, Per Knappskog, James A. Knowles, Nene Kobayashi, Karestan C. Koenen, Bettina Konte, Mayuresh Korgaonkar, Kaarina Kowalec, Marie-Odile Krebs, Mikael Landén, Claudine Laurent-Levinson, Lauren A. Lebois, Doug Levinson, Cathryn Lewis, Qingqin Li, Israel Liberzon, Greg Light, Sandra K. Loo, Yi Lu, Susanne Lucae, Charles Marmar, Nicholas G. Martin, Fermin Mayoral, Andrew M. McIntosh, Katie A. McLaughlin, Samuel A. McLean, Andrew McQuillin, Sarah E. Medland, Andreas Meyer-Lindenberg, Vihra Milanova, Philip B. Mitchell, Esther Molina, Bryan Mowry, Bertram Muller-Myhsok, Niamh Mullins, Robin Murray, Markus M. Nöthen, John I. Nurnberger, Kevin S. O’Connell, Roel A. Ophoff, Holly K. Orcutt, Michael J. Owen, Aarno Palotie, Carlos Pato, Michele Pato, Joanna Pawlak, Triinu Peters, Tracey L. Petryshen, Giorgio Pistis, James B. Potash, John Powell, Martin Preisig, Digby Quested, Josep A. Ramos-Quiroga, Andreas Reif, Kerry J. Ressler, Marta Ribasés, Marcella Rietschel, Victoria B. Risbrough, Margarita Rivera, Alex O. Rothbaum, Barbara O. Rothbaum, Dan Rujescu, Takeo Saito, Alan R. Sanders, Russell J. Schachar, Peter R. Schofield, Eva C. Schulte, Thomas G. Schulze, Laura J. Scott, Soraya Seedat, Christina Sheerin, Jianxin Shi, Pamela Sklar, Susan Smalley, Olav B. Smeland, Jordan W. Smoller, Edmund Sonuga-Barke, David St. Clair, Nils Eiel Steen, Dan Stein, Frederike Stein, Murray B. Stein, Fabian Streit, Neal Swerdlow, Florence Thibaut, Johan H. Thygesen, Ilgiz Timerbulatov, Claudio Toma, Edward Trapido, Micheline Tremblay, Ming T. Tsuang, Monica Uddin, Marquis P. Vawter, John B. Vincent, Henry Völzke, James T. Walters, Cynthia S. Weickert, Lauren A. Weiss, Myrna M. Weissman, Thomas Werge, Stephanie H. Witt, Miguel Xavier, Robert Yolken, Ross M. Young, Tetyana Zayats, Lori A. Zoellner, AGP Consortium, PEIC Psychosis Endophenotypes International Consortium, ADHD Working Group of the Psychiatric Genomics Consortium, Autism Working Group of the Psychiatric Genomics Consortium, Bipolar Disorder Working Group of the Psychiatric Genomics Consortium, Major Depressive Disorder Working Group of the Psychiatric Genomics Consortium, PTSD Working Group of the Psychiatric Genomics Consortium, Schizophrenia Working Group of the Psychiatric Genomics Consortium, CNV Working Group of the Psychiatric Genomics Consortium, Kimberley Kendall, Brien Riley, Naomi R. Wray, Michael C. O’Donovan, Patrick F. Sullivan, Sandra Sanchez-Roige, Caroline M. Nievergelt, Sébastien Jacquemont, Stephen W. Scherer, Jonathan Sebat

**Affiliations:** 1 The Centre for Applied Genomics, The Hospital for Sick Children, Toronto, ON, Canada,; 2 Program in Genetics and Genome Biology, The Hospital for Sick Children, Toronto, ON, Canada,; 3 Bioinformatics and Systems Biology Graduate Program, University of California San Diego, La Jolla, CA, USA,; 4 Department of Psychiatry, University of California San Diego, La Jolla, CA, USA,; 5 Centre Hospitalier Universitaire Sainte-Justine Research Center, Montreal, QC, Canada,; 6 Donders Institute for Brain, Cognition and Behaviour, Radboud University Medical Center, Nijmegen, The Netherlands,; 7 Department of Human Genetics, Radboud University Medical Center, Nijmegen, The Netherlands,; 8 Veterans Affairs San Diego Healthcare System, Center of Excellence for Stress and Mental Health, San Diego, CA, USA,; 9 Veterans Affairs San Diego Healthcare System, Research Service, San Diego, CA, USA,; 10 CHU Sainte-Justine Azrieli Research Center, Université de Montréal, Montreal, QC, Canada,; 11 Institute of Medical Science, University of Toronto, Toronto, ON, Canada,; 12 Tahoe Therapeutics (formerly Vevo), South San Francisco, CA, USA,; 13 Department of Molecular Genetics, University of Toronto, Toronto, ON, Canada,; 14 Molecular Medicine Program, The Hospital for Sick Children, Toronto, ON, Canada,; 15 KG Jebsen Centre for Neurodevelopmental disorders, University of Oslo, Oslo, Norway,; 16 Centre for Precision Psychiatry, University of Oslo, Oslo, Norway,; 17 Department of Medical Genetics, Oslo University Hospital, Oslo, Norway,; 18 Centre for Clinical Brain Sciences, University of Edinburgh, Edinburgh, UK,; 19 Department of Clinical Science, Umeå University, Umeå, Sweden,; 20 Institute of Clinical Medicine, University of Oslo, Oslo, Norway,; 21 Centre for Psychiatry Research, Department of Clinical Neuroscience, Karolinska Institutet & Stockholm Health Care Services, Stockholm Region, Stockholm, Sweden,; 22 Department of Psychiatric Research, Diakonhjemmet Hospital, Oslo, Norway,; 23 Department of Epidemiology, Columbia University, Robert N Butler Columbia Aging Center, New York, NY, US,; 24 National Institute of Mental Health, Klecany, Czech Republic,; 25 Department of Psychiatry, Dalhousie University, Halifax, NS, Canada,; 26 Division of Psychiatry, University of Edinburgh, Edinburgh, UK,; 27 Department of Psychiatry, Virginia Institute for Psychiatric and Behavioral Genetics, Virginia Commonwealth University, Richmond, VA, USA,; 28 Department of Neurology, Klinikum rechts der Isar, School of Medicine, Technical University of Munich, Munich, Germany,; 29 Division of Mental Health and Addiction, Oslo University Hospital, Oslo, Norway,; 30 Biomedical Network Research Centre on Mental Health (CIBERSAM), Madrid, Spain,; 31 Department of Mental Health, Hospital Universitari Vall d’Hebron, Barcelona, Spain,; 32 Department of Genetics, Microbiology, and Statistics, Faculty of Biology, Universitat de Barcelona (UB), Barcelona, Spain,; 33 Psychiatric Genetics Unit, Group of Psychiatry, Mental Health and Addiction, Vall d’Hebron Research Institute (VHIR), Universitat Autònoma de Barcelona, Barcelona, Spain,; 34 Boston Children’s Hospital, Division of Adolescent and Young Adult Medicine, Boston, MA, USA,; 35 Department of Pediatrics, Harvard Medical School, Boston, MA, USA,; 36 Department of Social and Behavioral Sciences, Harvard T.H. Chan School of Public Health, Boston, MA, USA,; 37 University College London, London, UK,; 38 Division of Psychiatry, University College London, London, UK,; 39 Department of Psychiatry, University of Münster, Münster, Germany,; 40 The Flore Institute of Neuroscience and Mental Health, Melbourne, Australia,; 41 Department of Psychiatry, University of Melbourne, Melbourne, Australia,; 42 Department of Psychiatry, Psychosomatic Medicine and Psychotherapy; University Hospital Frankfurt - Goethe University, Frankfurt, Germany,; 43 Institute of Epidemiology and Social Medicine, University of Münster, Münster, Germany,; 44 Department of Quantitative Health Sciences, Mayo Clinic, Rochester, MN, USA,; 45 Department of Psychiatry and Psychology, Mayo Clinic, Rochester, MN, USA,; 46 Department of Psychiatry and Behavioral Sciences, Institute for Genomics in Health, State University of New York Downstate Health Sciences University, Brooklyn, NY, USA,; 47 Cardiff University, National Centre for Mental Health, MRC Centre for Psychiatric Genetics and Genomics, Cardiff, UK,; 48 University Medical Center, Division of Neurosciences, Department of Psychiatry, Heidelberglaan, Utrecht, the Netherlands,; 49 Social, Genetic and Developmental Psychiatry Centre, Institute of Psychiatry, Psychology and Neuroscience, King’s College London, London, UK,; 50 Department of Translational Research in Psychiatry, Max Planck Institute of Psychiatry, Munich, Germany,; 51 School of Psychology, Faculty of Science, University of New South Wales, Sydney, New South Wales, Australia,; 52 Department of Nutrition, University of North Carolina at Chapel Hill, Chapel Hill, NC, USA,; 53 Department of Medical Epidemiology and Biostatistics, Karolinska Institutet, Sweden,; 54 Department of Psychiatry, University of North Carolina at Chapel Hill, Chapel Hill, NC, USA,; 55 Seaver Autism Center, Department of Psychiatry, Icahn School of Medicine at Mount Sinai, New York, NY, USA,; 56 Precision Medicine Research Program, Hunter Medical Research Institute, Newcastle, New South Wales, Australia,; 57 School of Biomedical Sciences and Pharmacy, University of Newcastle, Callaghan, NSW, Australia,; 58 Chronic Diseases Research Centre (CEDOC), Lisbon Institute of Global Mental Health, Lisbon, Portugal,; 59 Department of Psychiatry and Neuroscience Institute, University of Cape Town, Cape Town, South Africa,; 60 INSERM EPI 9906, Faculté de Médecine et de Pharmacie, Institut Fédératif de Recherches Multidisciplinaires sur les peptides, Rouen, France,; 61 Department of Psychiatry, Monash University, Melbourne, Australia,; 62 School of Psychiatry, University of New South Wales, Sydney, New South Wales, Australia,; 63 Psychiatric Epidemiology and Psychopathology Research Center, Department of Psychiatry, Lausanne University Hospital and University of Lausanne, Prilly, Switzerland,; 64 Université Paris Cité, Institute of Psychiatry and Neuroscience of Paris (INSERM U1266), GHU Paris Psychiatrie et Neurosciences, Paris, France,; 65 Institute of Human Genetics, University of Bonn, School of Medicine & University Hospital Bonn, Bonn, Germany,; 66 Department of Child and Adolescent Psychiatry, Pitié-Salpêtrière Hospital, Assistance Publique-Hôpitaux de Paris-Sorbonne University, Paris, France,; 67 CNRS UMR 7222, Institute for Intelligent Systems and Robotics, Sorbonne University, Paris, France,; 68 Department of Psychiatry, Trinity College Dublin, Dublin, Ireland,; 69 Centre for Neuropsychiatric Genetics and Genomics, Division of Psychological Medicine and Clinical Neurosciences, Cardiff University, Cardiff, UK,; 70 Department of Psychiatry, University of Toronto, Toronto, ON, Canada,; 71 Neurosciences & Mental Health, The Hospital for Sick Children, Toronto, ON, Canada,; 72 Department Genes and Environment, Max-Planck-Institute of Psychiatry, Munich, Germany,; 73 Joint Institute for Individualisation in a Changing Environment (JICE), University of Münster and Bielefeld University, Münster, Germany,; 74 Institute for Translational Psychiatry, University of Münster, Münster, Germany,; 75 Department of Psychological Sciences, Kent State University, Kent, OH, USA,; 76 Institute of Medical Informatics and Statistics, UKSH University Hospital of Schleswig-Holstein Kiel Campus, Arnold-Heller-Strasse, Kiel, Germany,; 77 Institute for Human Genetics, Department of Psychiatry and Behavioral Sciences, Weill Institute for Neurosciences, University of California San Francisco, San Francisco, CA, USA,; 78 Data Science & Biometrics, Research & Development, Pierre Fabre Group, Toulouse, France,; 79 School of Medicine, Division of Psychological Medicine and Clinical Neurosciences, Cardiff University, Cardiff, UK,; 80 Sheppard Pratt Health System, Baltimore, MD, USA,; 81 Department of Medical Genetics, Oslo University Hospital and University of Oslo, Oslo, Norway,; 82 Centre for Precision Psychiatry, Oslo University Hospital & University of Oslo, Oslo, Norway,; 83 Department of Psychiatry and Psychotherapy, University of Freiburg, Freiburg, Germany,; 84 Cheshire and Wirral Partnership NHS Trust, Ellesmere Port, Cheshire, UK,; 85 Department of Psychiatry, Sørlandet hospital, Arendal/Kristiansand, Norway,; 86 Department of Psychiatry and Behavioral Sciences, Stanford University, Stanford, CA, USA,; 87 Wu Tsai Neurosciences Institute, Stanford University, Stanford, CA, USA,; 88 Department of Biochemistry & Molecular Biology, Indiana University, Indianapolis, IN, USA,; 89 Department of Medical and Molecular Genetics, Indiana University, Indianapolis, IN, USA,; 90 Estonian Genome Centre, Institute of Genomics, University of Tartu, Tartu, Estonia,; 91 Departments of Psychiatry and of Neuroscience and Physiology, SUNY Upstate Medical University, Syracuse, New York, NY, USA,; 92 Department of Psychological Sciences, Case Western Reserve University, Cleveland, OH, USA,; 93 Institute of Medical Genetics and Pathology, University Hospital Basel, Basel, Switzerland,; 94 Centre for Human Genetics, University of Marburg, Marburg, Germany,; 95 Institute of Human Genetics, University of Bonn School of Medicine & University Hospital Bonn, Bonn, Germany,; 96 Department of Biomedicine, University of Basel, Basel, Switzerland,; 97 Department of Psychiatry (UPK), University of Basel, Basel, Switzerland,; 98 Department of Medical Neuroscience, Donders Institute of Brain, Cognition and Behaviour, Radboud University Medical Center, Nijmegen, The Netherlands,; 99 Department of Human Genetics, Donders Institute of Brain, Cognition and Behaviour, Radboud University Medical Center, Nijmegen, The Netherlands,; 100 Department of Psychiatry and Psychology, Mayo Clinic, Rochester, MN, US,; 101 Janssen Research and Development, LLC,; 102 School of Biomedical Sciences, University of New South Wales, Sydney, New South Wales, Australia,; 103 Neuroscience Research Australia, Randwick, New South Wales, Australia,; 104 FSBSI Institute of Biochemistry and Genetics of the Ufa Federal Research Center of the Russian Academy of Sciences, Russia,; 105 FSBEI HE Bashkir State Medical University of Health Ministry of Russia, Russia,; 106 FSBEI APGE Russian Medical Academy of Continuing Professional Education of the Ministry of Health of Russia, Russia,; 107 FSBEI HE Kemerovo State University, Russia,; 108 Department of Psychiatry and Psychotherapy, University Medicine Greifswald, Greifswald, Germany,; 109 Department of Psychiatry and Behavioral Neuroscience, University of Chicago, Chicago, IL, USA,; 110 School of Medicine, University of California Los Angeles, Los Angeles, CA, USA,; 111 Department of Psychiatry and Psychotherapy, Comprehensive Center for Clinical Neurosciences and Mental Health (C3NMH), Medical University of Vienna, Austria,; 112 Director, Psychiatric Genetic Epidemiology & Neurobiology Laboratory (PsychGENe Lab), SUNY Upstate Medical University,; 113 Division of Human Genetics, Children’s Hospital of Philadelphia, Philadelphia, PA, USA,; 114 Center for Applied Genomics, Children’s Hospital of Philadelphia, Philadelphia, PA, USA,; 115 Department of Pediatrics, Perelman School of Medicine, University of Pennsylvania, Philadelphia, PA, USA,; 116 Department of Psychiatry and Behavioral Sciences, Johns Hopkins University School of Medicine, Baltimore, MD, USA,; 117 Psychological Medicine, University of Worcester, Worcester, UK,; 118 Discipline of Psychiatry and Mental Health, School of Clinical Medicine, Faculty of Medicine and Health, University of New South Wales, Sydney, New South Wales, Australia,; 119 Center on Enhancement of Community Integration for Homeless Veterans, VA Greater Los Angeles Healthcare System, Los Angeles, CA, USA,; 120 Semel Institute for Neuroscience and Human Behavior, University of California Los Angeles, Los Angeles, CA, USA,; 121 Psychiatric Genetics Research Unit, Alexandru Obregia Clinical Psychiatric Hospital, Bucharest, Romania,; 122 Department of Psychiatry, University of Pennsylvania, Philadelphia, PA, USA,; 123 Unidad de Gestión Clínica de Salud Mental del Hospital Regional Universitario de Málaga, Instituto de Investigación Biomédica de Málaga y Plataforma en Nanomedicina - IBIMA Plataforma Bionand, Málaga, Spain,; 124 JanK.G. Jebsen Centre for Neuropsychiatric Disorders, Department of Biomedicine, University of Bergen, Bergen, Norway,; 125 Department of Psychiatry and Behavioral Sciences (JKF, PL, BJ, MWM, JH, JY), Stanford University School of Medicine, Stanford, CA, USA,; 126 VISN 21 Mental Illness Research, Education, and Clinical Center (JKF, PL, MWM, JH, JY), Veterans Affairs Palo Alto Health Care System, Palo Alto, CA, USA,; 127 Medical Research Council Centre for Neuropsychiatric Genetics and Genomics, Division of Psychological Medicine and Clinical Neurosciences, Cardiff University, Cardiff, UK,; 128 Department of Psychiatry and Psychotherapy, Medical University of Vienna, Austria,; 129 University of Peshawar, Peshawar, Pakistan,; 130 MRC Human Genetics Unit, Institute of Genetics and Molecular Medicine, Edinburgh, UK,; 131 Department of Child and Adolescent Psychiatry, Psychotherapy and Psychosomatics, University Hospital Essen (AöR), University of Duisburg-Essen, Essen, Germany,; 132 Center for Translational Neuro- and Behavioral Sciences, University Hospital Essen, University of Duisburg-Essen, Essen, Germany,; 133 Faculty of Medicine and Health Sciences, Department of Psychiatry, Stellenbosch University, Cape Town, Western Cape, ZA,; 134 SAMRC Genomics of Brain Disorders Research Unit, Stellenbosch University, Cape Town, Western Cape, ZA,; 135 Department of Genetic Epidemiology, Institute of Human Genetics, University of Münster, Münster, Germany,; 136 Section of Molecular Genetics of Mental Disorders, LVR-University Clinic Essen, Essen, Germany,; 137 Institute of Sex and Gender-Sensitive Medicine, University Hospital Essen, University of Duisburg-Essen, Virchowstr, Essen, Germany,; 138 Interfaculty Institute for Genetics and Functional Genomics, University Medicine Greifswald, Greifswald, Germany,; 139 Institute of Biological Psychiatry, Mental Health Services, Copenhagen University Hospital, Roskilde, Denmark,; 140 Charles Bronfman Institute for Personalized Medicine, Icahn School of Medicine at Mount Sinai, New York, NY, USA,; 141 Laboratory of Integrative Neuroscience, Universidade Federal de São Paulo, São Paulo, Brazil,; 142 Department of Biochemistry, Universidade Federal de São Paulo, São Paulo, Brazil,; 143 Department of Psychiatry, Icahn School of Medicine at Mount Sinai, New York, NY, USA,; 144 Department of Genetics and Genomic Sciences, Icahn School of Medicine at Mount Sinai, New York, NY, USA,; 145 Department of Psychiatry, Fujita Health University School of Medicine, Toyoake, Aichi, Japan,; 146 Cardiff University, National Centre for Mental Health, Cardiff University Centre for Psychiatric Genetics and Genomics, Cardiff, UK,; 147 Institute of Neuroscience and Physiology, University of Gothenburg, Gothenburg, Sweden,; 148 Department of Medical Epidemiology and Biostatistics, Karolinska Institutet, Stockholm, Sweden,; 149 McLean Hospital, Belmont, MA, USA,; 150 Department of Psychiatry, Harvard Medical School, Boston, MA, USA,; 151 Campbell Family Mental Health Research Institute, Centre for Addiction and Mental Health, Toronto, ON, Canada,; 152 The Ohio State University, College of Medicine, Institute for Behavioral Medicine Research, Columbus, OH, USA,; 153 Department of Psychiatry and Psychotherapy, University of Marburg, Marburg, Germany,; 154 Center for Mind, Brain and Behavior, University of Marburg, Marburg, Germany,; 155 Department of Psychological Medicine and Neurology, MRC Centre for Neuropsychiatric Genetics and Genomics, School of Medicine, Neuroscience and Mental Health Research Institute, Cardiff University, Cardiff, UK,; 156 Department of Clinical Science, University of Bergen, Bergen, Norway,; 157 Department of Medical Genetics, Haukeland University Hospital, Bergen, Norway,; 158 Department of Genetics, Rutgers University, Piscataway, NJ, USA,; 159 Human Genetics Institute of New Jersey (HGINJ), Rutgers University, Piscataway, NJ, USA,; 160 Goethe University Frankfurt, University Hospital, Department of Psychiatry, Psychosomatic Medicine and Psychotherapy, Frankfurt, Germany,; 161 Department of Epidemiology, Harvard T. H. Chan School of Public Health, Boston, MA, USA.,; 162 Brain Dynamics Centre, Westmead Institute for Medical Research, University of Sydney, Sydney, New South Wales, Australia,; 163 Childhood Genetic Disease Laboratory, INSERM UMR S933, Trousseau University Hospital, Paris, France,; 164 McLean Hospital, Center of Excellence in Depression and Anxiety Disorders, Belmont, MA, USA,; 165 Department of Medical and Molecular Genetics, Faculty of Life Sciences and Medicine, King’s College London, London, UK,; 166 Department of Psychiatry and Behavioral Sciences, Texas A&M University College of Medicine, Bryan, TX, USA,; 167 Department of Psychiatry and Biobehavioral Sciences, University of California Los Angeles, Los Angeles, CA, USA,; 168 College of Pharmacy, University of Manitoba, Winnipeg, MB, Canada,; 169 New York University, Grossman School of Medicine, New York City, NY, USA,; 170 Brain and Mental Health Program, QIMR Berghofer Institute of Medical Research, Brisbane, Australia,; 171 Division of Psychiatry, Centre for Clinical Brain Sciences, The University of Edinburgh, Edinburgh, UK,; 172 Department of Psychology, Harvard University, Boston, MA, USA,; 173 Department of Emergency Medicine, UNC Institute for Trauma Recovery, Chapel Hill, NC, USA,; 174 Department of Anesthesiology, UNC Institute for Trauma Recovery, Chapel Hill, NC, USA,; 175 School of Psychology, The University of Queensland, Brisbane, Queensland, Australia,; 176 School of Psychology and Counselling, Queensland University of Technology, Brisbane, Queensland, Australia,; 177 Mental Health and Neuroscience, QIMR Berghofer Medical Research Institute, Brisbane, Queensland, Australia,; 178 Department of Psychiatry and Psychotherapy, Central Institute of Mental Health, Medical Faculty Mannheim, Heidelberg University, Mannheim, Germany,; 179 Department of Psychiatry, Faculty of Medicine, Medical University of Sofia, University Hospital Alexandrovska, Bulgaria,; 180 Department of Nursing, Faculty of Health Sciences, University of Granada, Granada, Spain.,; 181 Institute of Neurosciences ‘Federico Olóriz’, Biomedical Research Centre (CIBM), University of Granada, and Instituto de Investigación Biosanitaria, Ibs Granada, Granada, Spain.,; 182 Queensland Centre for Schizophrenia Research, Wolston Park Hospital, Wacol, Queensland, Australia,; 183 Department of Psychiatry, University of Queensland, Brisbane, Australia,; 184 HMNC Holding GmbH, Munich, Germany,; 185 Max Planck Institute of Psychiatry, Munich, Germany,; 186 Department of Psychosis Studies, Institute of Psychiatry, Psychology and Neuroscience,King’s College London, UK,; 187 Departments of Psychiatry & Medical and Molecular Genetics, Indiana University School of Medicine, Indianapolis, IN, USA,; 188 Stark Neurosciences Research Institute,; 189 Department of Psychiatry and Biobehavioral Science and Department of Human Genetics, David Geffen School of Medicine, University of California Los Angeles, Los Angeles, CA, USA,; 190 Department of Psychology, Northern Illinois University, DeKalb, IL, USA,; 191 Centre for Neuropsychiatric Genetics and Genomics, Division of Psychiatry and Clinical Neurosciences, Cardiff University, Hadyn Ellis Building, Maindy Road, Cardiff, CF24 4HQ,; 192 Analytic and Translational Genetics Unit, Department of Medicine, Department of Neurology, and Department of Psychiatry, Massachusetts General Hospital, Boston, MA, USA,; 193 The Stanley Center for Psychiatric Research and Program in Medical and Population Genetics, The Broad Institute of MIT and Harvard, Cambridge, MA, USA,; 194 Institute for Molecular Medicine Finland and the Helsinki Institute of Life Science, University of Helsinki, Helsinki, Finland,; 195 Department of Psychiatry, Rutgers University, Piscataway, NJ, USA,; 196 Department of Psychiatric Genetics, Poznan University of Medical Sciences, Poznan, Poland,; 197 Psychiatric and Neurodevelopmental Genetics Unit, Department of Psychiatry and Center for Genomic Medicine, Massachusetts General Hospital, Harvard Medical School, Boston, MA, USA,; 198 King’s College London, London, UK,; 199 Oxford NHS Foundation Trust, Oxford, UK,; 200 Department of Psychiatry, Psychosomatic Medicine and Psychotherapy, University Hospital Frankfurt - Goethe University, Frankfurt am Main, Germany,; 201 Department of Psychiatry and Behavioral Sciences, Emory University, Atlanta, GA, USA,; 202 Division of Depression and Anxiety, McLean Hospital, Belmont, MA, US,; 203 Department of Genetic Epidemiology in Psychiatry, Central Institute of Mental Health, Medical Faculty Mannheim, Heidelberg University, Mannheim, Germany,; 204 Department of Biochemistry and Molecular Biology II, Faculty of Pharmacy, University of Granada, Granada, Spain,; 205 Department of Research and Outcomes, Skyland Trail, Atlanta, GA, USA,; 206 Department of Psychiatry and Behavioral Neuroscience, University of Chicago,; 207 German Center for Mental Health (DZPG), Munich, Germany,; 208 Department of Psychiatry, University Hospital, Faculty of Medicine, University of Bonn, Bonn, Germany,; 209 Institute of Psychiatric Phenomics and Genomics (IPPG), LMU University Hospital, LMU Munich, Munich, Germany,; 210 Department of Psychiatry and Psychotherapy, LMU University Hospital, LMU Munich, Munich, Germany,; 211 Department of Biostatistics and Center for Statistical Genetics, School of Public Health, University of Michigan, Ann Arbor, MI, USA,; 212 SAMRC Extramural Genomics of Brain Disorders Research Unit, Stellenbosch University, Cape Town, Western Cape, ZA,; 213 SAMRC Unit on the Genomics of Brain Disorders, Faculty of Medicine and Health Sciences, Department of Psychiatry, Stellenbosch University, Cape Town, Western Cape, ZA,; 214 Division of Cancer Epidemiology and Genetics National Cancer Institute, Rockville, MD, USA,; 215 Icahn School of Medicine at Mount Sinai, New York, NY, USA,; 216 Net.bio Inc, Los Angeles, CA, USA,; 217 Psychiatric and Neurodevelopmental Genetics Unit, Center for Genomic Medicine, Massachusetts General Hospital, Boston, MA, USA,; 218 Center for Precision Psychiatry, Department of Psychiatry, Massachusetts General Hospital, Boston, MA, USA,; 219 Department of Child and Adolescent Psychiatry, Institute of Psychiatry, Psychology and Neuroscience, King’s College London, London, UK,; 220 Department of Mental Health, University of Aberdeen, Royal Cornhill Hospital, Aberdeen, UK.,; 221 Division of Mental Health and Substance abuse, Diakonhjemmet Hospital, Oslo, Norway,; 222 SAMRC Unit on Risk and Resilience in Mental Disorders, Dept of Psychiatry and Neuroscience Institute, University of Cape Town, Cape Town, South Africa,; 223 School of Public Health, University of California San Diego, La Jolla, CA, USA,; 224 Veterans Affairs San Diego Healthcare System, Psychiatry Service, San Diego, CA, USA,; 225 German Center for Mental Health (DZPG), Partner Site Mannheim - Heidelberg - Ulm, Germany,; 226 Hector Institute for Artificial Intelligence in Psychiatry, Central Institute of Mental Health, Medical Faculty Mannheim, Heidelberg University, Mannheim, Germany,; 227 Cochin University Hospital, Paris Cité University, Paris, France,; 228 INSERM Unit 894, Institute of Psychiatry And Neuroscience of Paris, Paris, France,; 229 Institute for Health Informatics, University College London, London, UK,; 230 SBI MD Central Clinical Psychiatric Hospital F. Usoltseva,; 231 FSBEI HE “Russian University of Medicine” of the Ministry of Health of Russia, Russia,; 232 Neuroscience Research Australia, Sydney, New South Wales, Australia,; 233 School of Biomedical Sciences, Faculty of Medicine and Health, University of New South Wales, Sydney, New South Wales, Australia,; 234 School of Public Health and Department of Epidemiology, Louisiana State University Health Sciences Center, New Orleans, LA, US,; 235 Cheshire and Wirral Partnership NHS Foundation Trust, Winsford, Cheshire, UK,; 236 Genomics Program, University of South Florida College of Public Health, Tampa, FL, USA,; 237 Department of Psychiatry & Human Behavior, University of California, Irvine, CA, USA,; 238 Institute for Community Medicine, University Medicine Greifswald, Greifswald, Germany,; 239 New York State Psychiatric Institute, New York, NY, USA,; 240 Department of Psychiatry, Columbia University Vagelos College of Physicians and Surgeons, New York, NY, USA,; 241 Institute of Biological Psychiatry, Mental Health Services, Copenhagen University Hospital, Copenhagen, Denmark,; 242 Universidade Nova de Lisboa, Nova Medical School, Lisboa, Portugal,; 243 Stanley Division of Developmental Neurovirology, Johns Hopkins School of Medicine, Baltimore, MD, USA,; 244 University of the Sunshine Coast, The Chancellory, Sippy Downs, Queensland, Australia,; 245 Queensland University of Technology, School of Clinical Sciences, Kelvin Grove, Queensland, Australia,; 246 Department of Biomedicine, University of Bergen, Bergen, Norway,; 247 Stanley Center for Psychiatric Research, Broad Institute of MIT, and Harvard, Cambridge, MA, USA,; 248 Analytic and Translational Genetics Unit, Department of Medicine, Massachusetts General Hospital, and Harvard Medical School, Boston, MA, USA,; 249 Department of Psychology, University of Washington, Seattle, WA, USA,; 250 Department of Psychiatry, Virginia Institute for Psychiatric and Behavioral Genetics, Virginia Commonwealth University, Richmond, VA, USA,; 251 Department of Psychiatry, University of Oxford, Warneford Hospital, Oxford, UK,; 252 Institute for Molecular Bioscience, The University of Queensland, St Lucia, Queensland, Australia,; 253 Departments of Genetics and Psychiatry, University of North Carolina, Chapel Hill, NC, USA,; 254 Institute for Genomic Medicine, University of California San Diego, La Jolla, CA, USA,; 255 Department of Medicine, Division of Genetic Medicine, Vanderbilt University, Nashville, TN, USA,; 256 Research/Psychiatry, Veterans Affairs San Diego Healthcare System, San Diego, CA, USA,; 257 Department of Pediatrics, Université de Montréal, Montreal, QC, Canada,; 258 McLaughlin Centre, University of Toronto, Toronto, ON, Canada,; 259 Beyster Center for Psychiatric Genomics, University of California San Diego, La Jolla, CA, USA,; 260 Department of Cellular and Molecular Medicine, University of California San Diego, La Jolla, CA, USA

## Abstract

Psychiatric conditions share common genes, but mechanisms that differentiate diagnoses remain unclear. We present a multidimensional framework for functional analysis of rare copy number variants (CNVs) across 6 diagnostic categories, including schizophrenia (SCZ), autism (ASD), bipolar disorder (BD), depression (MDD), PTSD, and ADHD (N = 574,965). Using gene-set burden analysis (GSBA), we tested duplication (DUP) and deletion (DEL) burden across 2,645 functional gene sets defined by the intersections of pathways, cell types, and cortical regions. While diagnoses converge on shared pathways, mixed-effects modeling revealed divergence of pathway effects by cell type, brain region, and gene dosage. Factor analysis identified latent dimensions aligned with clinical axes. A primary factor (F1) captured reciprocal dose-dependent effects of DUP and DEL in SCZ reflecting positive and negative effects in excitatory versus inhibitory neurons and association versus sensory cortex. SCZ and ASD were both strongly aligned with F1 but with opposing directionalities. Orthogonal factors highlighted neuronal versus non-neuronal effects in mood disorders (F2) and differential spatial distributions of DEL effects in ADHD and MDD (F3). High-impact CNVs at 16p11.2 and 22q11.2 were enriched for combinations of cell-type-specific genes involved in pathways consistent with our broader findings. These results reveal molecular and cellular mechanisms that are broadly shared across psychiatric traits but differ between diagnostic categories in context and directionality.

## Background

Genes that are associated with psychiatric conditions carry rich information about the timing, location, and nature of the biological processes that contribute to psychopathology^1,2^. The molecular functions of genes point to the cellular pathways and regulatory networks that underlie vulnerability to psychiatric disorders. Furthermore, because gene expression is tightly regulated in a cell-type and region-specific manner across the brain, the discovery of genes can also provide insight into the neuroanatomical circuits that influence psychiatric traits. The discovery of hundreds of genes and copy number variations (CNVs) that underlie major psychiatric conditions such as schizophrenia (SCZ)^3–6^ and autism spectrum disorder (ASD)^7–11^ has implicated a variety of pathways including synaptic function, chromatin regulation, cell signaling, cytoskeletal proteins, and DNA and RNA binding proteins that regulate neurodevelopment^3,12–18^. Similar pathways have been implicated by transcriptome characterization of post-mortem brains from case samples of idiopathic ASD, SCZ and bipolar disorder (BD)^19–23^. Genes implicated in psychiatric diagnoses are also enriched in specific neural cell types. RNA sequencing in postmortem samples have identified neuronal and glial signatures associated with ASD^24,25^ and differences in the distributions of glial and neuronal cells in mood disorders^26^. Analysis of GWAS associations has found enrichment of SCZ^27^, major depressive disorder (MDD) and post-traumatic stress disorder (PTSD)^28^ associations in mature excitatory and inhibitory neurons.

Despite significant progress in identifying risk genes and pathways in psychiatric conditions, there remains a limited understanding of how neural processes relate to specific psychiatric traits or diagnoses. Many of the same biological pathways, such as those described above, have been repeatedly associated with multiple diagnostic categories, including SCZ^5,15,29^, BD^14,30^, ASD^16^, intellectual disability^31^ and congenital heart disease^32^. Thus, functional convergence that is evident from pathway enrichment analysis of the associated genes highlights broad biological themes but lacks the resolution to differentiate neural mechanisms that differ between diagnostic categories.

**Fig. 1|:**
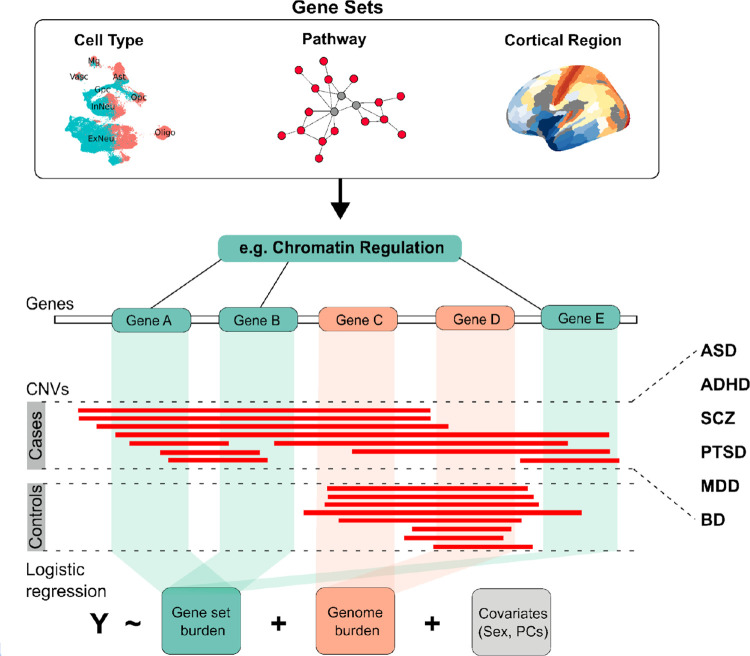
Investigating association of pathways, cell types and brain regions by Gene Set Burden Analysis (GSBA). Gene sets were derived for Pathway (from GO, KEGG, REACTOME, and BioCarta), Cell type (from single cell study, Velmeshev et al.), and Cortical regions (from Glasser parcellation of the Allen Brain Atlas). Case-control association of CNV burden collapsed across gene sets, was then tested by logistic regression and meta-analysis was performed across genotyping platforms. Functional gene set associations were tested for 6 major psychiatric conditions (ASD, ADHD, SCZ, PTSD, MDD, BD).

CNVs have been shown to exert dose-dependent effects on a range of complex traits, including gene expression^33^, head size^4,34^, brain volume^35,36^, functional connectivity^37^, body mass^38^, craniofacial morphology^39^. As described in our companion paper^40^, this pattern extends to psychiatric traits, where reciprocal duplications (DUPs) and deletions (DELs) of genes show dose-dependent effects and diverge in their genotype-phenotype associations. A more detailed functional analysis of gene-dosage effects could clarify how alterations in molecular pathways contribute to psychiatric traits. In this study, we developed and applied an integrated framework to examine how gene-dosage effects on pathways, cell types, and brain regions relate to clinical diagnoses (Fig. 1). Key elements of this approach include accounting for (1) directionality of gene-dosage effects and their distribution within (2) neural cell-types and (3) cortical brain regions, and we perform a comparative analysis across multiple diagnostic categories.

### Gene set association of rare CNVs in 6 psychiatric conditions

We leveraged large-scale rare CNV data (population frequency <2%) from the Psychiatric Genomics Consortium, comprising genome-wide microarray data from 574,965 individuals (133,007 cases and 441,958 controls) across six major psychiatric disorders: schizophrenia (SCZ), autism spectrum disorder (ASD), bipolar disorder (BD), major depressive disorder (MDD), post-traumatic stress disorder (PTSD), and attention-deficit/hyperactivity disorder (ADHD). CNVs were uniformly processed through a centralized pipeline for calling and quality control. Only rare CNVs (frequency <2%) were retained for analysis. Individuals represented diverse ancestral backgrounds, with 89.3% of European ancestry. This dataset enabled us to apply our multidimensional framework to identify distinct molecular and cellular features of brain function associated with each psychiatric diagnosis.

We assembled a primary catalogue of 2,645 gene sets that capture neurobiological features across multiple levels of organization. These included 2,453 molecular pathways from public databases^41–43^. In addition, differential expression in single-cell expression data was analyzed to create gene sets for 12 cell types from human fetal and adult brain (ranging from second trimester to 54 years of age)^44^, and differential expression in bulk tissue was analyzed to create 180 anatomic regions of cerebral cortex from the Allen Human Brain Atlas (AHBA)^45 46^(Table S1).

We investigated the association of functional gene sets with psychiatric diagnoses using **gene-set burden analysis (GSBA)**^5^. Associations detected by GSBA capture the enrichment of variants in functionally-related genes in cases. However, GSBA is not equivalent to a gene-set enrichment test (e.g., Subramanian et al., 2005^47^). Rather, it is a statistical genetics approach that quantifies the effect size of rare-variant burden across a defined set of genes (e.g. a GO term) in cases and controls (Fig. 1). For each gene set, we tested the association of the aggregate DEL or DUP counts across genes with case-control status by logistic regression controlling for population structure, sex and overall genome-wide CNV burden (collapsed across all out-of-category genes). Gene-set summary statistics were generated for each genotyping platform in each diagnostic category, and results were combined by meta-analysis. Combined results were corrected for multiple testing with Benjamini-Hochberg False Discovery Rate (BH-FDR<5%, Fig. S1).

Significant functional burden associations were detected for a total of 787 gene sets in one or more conditions, including SCZ (671 gene sets) and ASD (331 gene sets), ADHD (52 gene sets), BD (122 gene sets) and MDD (3 gene sets) (Table S2). Comparing summary statistics between trans-ancestry analysis and the European-only subset, we found a high level of concordance in the z-statistics between single ancestry (European subset) and trans-ancestry results across the 6 psychiatric conditions (beta-coefficients are between 0.9 and 1 with median beta-coefficient = 0.97; Fig. S2). All results described below are from the trans-ancestry summary statistics, which has the greatest statistical power.

### Common neurodevelopmental pathways are implicated in multiple diagnostic categories

**Fig. 2|:**
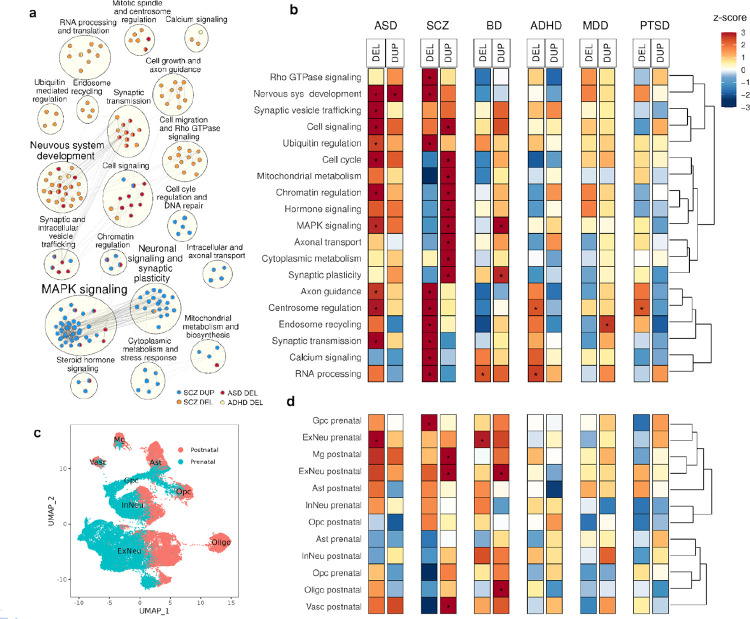
Rare CNVs association analysis results in molecular pathways and neuronal cell types. (**a**) Enrichment map showing clusters of functional modules that are significantly associated with any condition. CNV associations are color-coded as a portion with a node where red indicates a DEL association in ASD, orange indicates a DEL association in SCZ, blue indicates a DUP association in SCZ, and yellow indicates a DEL association in ADHD. Gene-sets not forming a cluster of 3 or more members were excluded. Gene set clusters are listed in Table S4. (**b**) The heatmap represents the results at the pathway-cluster level, with color indicating z-score from meta-analysis. (**c**) A UMAP plot displays cell clusters colored by prenatal (teal) and postnatal (red) periods. (**d**) Heatmaps show association results at the cell type level with color indicating z-score, where red represents a higher burden of CNVs in cases and blue represents a depletion of CNVs burden in cases. An asterisk indicates statistically significant associations (q-value <0.1). Summary statistics of the initial primary gene sets and for the final set of pathway clusters are in Tables S3, and S5 respectively.

Pathway gene sets were compiled from Gene Ontology (GO)^41^, KEGG^42^, Reactome^43^, and BioCarta^48^, with size ranging from 50 to 500 genes. 589 gene sets were associated with one or more conditions (Table S3). Using Enrichment Map^49^, overlapping gene sets implicated by CNVs were grouped into 19 functionally-related clusters representing canonical pathways such as MAPK signaling, nervous system development, synaptic transmission, chromatin regulation, etc. (Fig. 2a, Table S4). To summarize the pathway results, effect sizes were then estimated for the 19 gene sets in 6 diagnostic categories by GSBA regression (Fig. 2b, Table S5)

As expected, CNV burden associations were strongest in ASD and SCZ and were attenuated in other adult onset diagnoses, BD, ADHD, MDD, and PTSD. Many of the same functional gene sets were implicated in ASD and SCZ, including MAPK and other cell-signaling pathways, chromatin regulation, and synaptic transmission. Pathway signals in ASD were driven by significant DEL associations across 10 pathways. SCZ, by contrast, showed DUP associations in 9 functional gene sets such as chromatin regulation, MAPK signaling, axonal transport, and DEL associations in a different set of 9 pathways including synaptic transmission, axon guidance, and calcium signaling. The finding that pathway associations in SCZ differ by gene dosage is notable in light of the dose-dependent CNV effects reported for SCZ and other diagnoses in our companion study^40^.

### Gene set burden associations implicate neuronal and non-neuronal cell types

Twelve cell-type gene sets were derived from single-cell RNA-sequencing of human cortex (prefrontal, cingulate, insula, motor, and temporal regions) spanning prenatal (5–9 months) and postnatal (0–54 years of age) developmental stages, based on the dataset from Velmeshev et al.^44^. Starting from eight major cell type clusters defined in the original study, we refined these to capture key developmental distinctions, resulting in the following gene sets: five prenatal cell types - 1) glial progenitor cells (GpcPre), 2) oligodendrocyte precursor cells (OpcPre), 3) inhibitory neurons (InNeuPre), 4) excitatory neurons (ExNeuPre), and 5) astrocytes (AstPre); and seven postnatal cell types - 6) vascular cells (VascPost), 7) OpcPost, 8) oligodendrocytes (OligoPost), 9) microglia (MgPost), 10) inhibitory neurons (InNeuPost), 11) excitatory neurons (ExNeuPost), and 12) astrocytes (AstPost) (Fig. 2c). We observed several cell type associations with diagnostic categories (Fig. 2d, Table S3). ASD was associated with DEL burden in ExNeuPre, consistent with loss-of-function variants in ASD genes being enriched in fetal excitatory neurons^7,50^. SCZ showed DUP association in ExNeuPre, microglia, and neurovascular cells and DEL association in GpcPre. BD showed DUP association in ExNeuPost and OligoPost and DEL association in ExNeuPre.

### Diagnoses differ in the distribution of gene-set associations between sensorimotor and association cortex

Spatial variation in gene expression across the cortex reflects region-specific regulation beyond differences in cell type composition^51^. The primary gradient of gene expression (PC1) in the AHBA follows a sensorimotor-to-association (S-A) axis, spanning from primary sensory (visual, auditory, sensorimotor cortex) areas to transmodal (frontal, temporal) regions^51–53^. This axis aligns with several cortical hierarchies, including developmental timing^54–56^, myelination^54,55^, anatomical projections^57^, and functional specialization^58^. Given its close correspondence with the S-A axis^51^, we refer to AHBA PC1 as the S-A axis throughout the paper.

**Fig. 3|:**
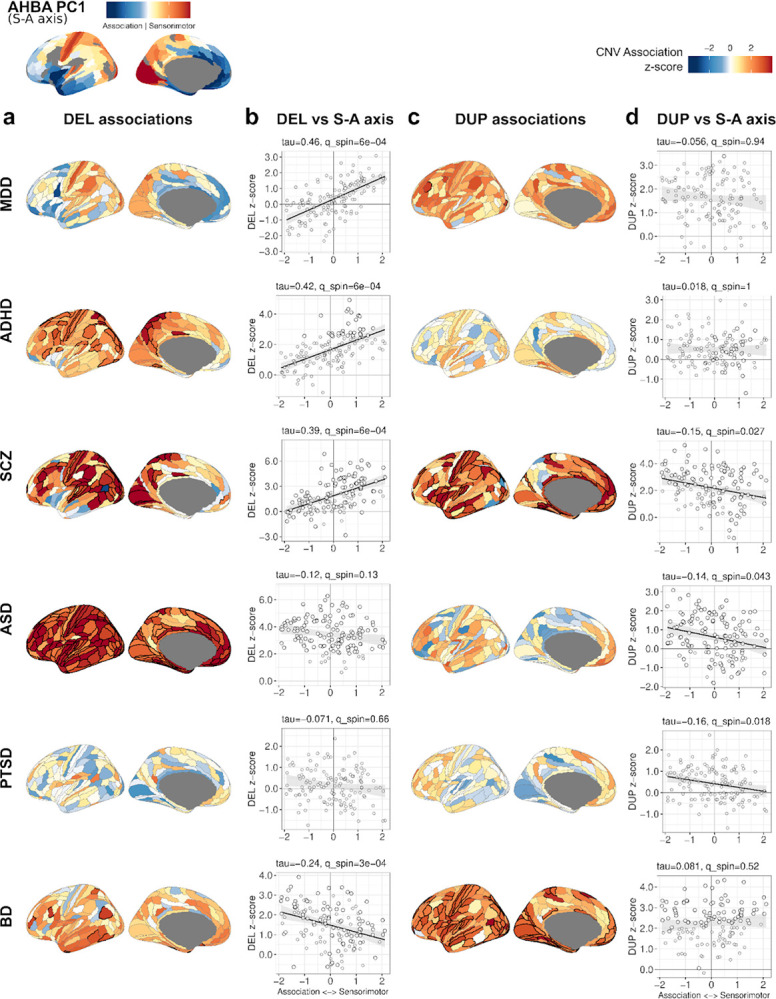
Rare (**a**) DEL and (**c**) DUP association analysis results of the cortical brain regions in the 6 conditions. Color indicates the association level (z-score) with red indicating the CNV association with the cases, while blue indicates the depletion of CNVs in cases (Table S3). Correlation results between CNV associations in (**b**) DEL and (**d**) DUP against the dominant transcriptomic brain gradient (PC1 of AHBA). Each circle represents a brain region gene set. Kendall’s Tau and corresponding q-value are shown in the title of each scatterplot. Solid diagonal trend line indicates significant correlation (q_SPIN_<0.05). The cortical map at the top left corner illustrates the transcriptomic gradient from PC1 AHBA.

To investigate how gene dosage effects are distributed across the cortex, we defined gene sets for each of the 180 cortical regions from Glasser et al.^45^. Gene expression was z-transformed across regions, and highly expressed genes (z>1) were assigned to each set of 180 regions. DEL and DUP burden was tested across cortical gene sets within each diagnosis. In total, 177 significant associations were identified. DEL and DUP associations are visualized on Glasser cortical maps (Fig. 3a, c), with effect sizes (z-scores) represented by a red-blue scale. We then tested whether spatial patterns of effect sizes aligned with the S-A axis using the SPIN test^59^ with 10,000 permutations and Kendall correlation.

CNV effect sizes varied across the cortex, and in several diagnostic categories, they showed significant, but divergent, correlations with the S-A axis. DEL effect sizes were positively correlated with the S-A axis in MDD, ADHD, and SCZ, indicating enrichment of DEL signal in sensorimotor cortex, while BD showed a negative correlation, indicating a relative enrichment of DEL signal in association cortex (Fig. 3b; Table S3). DUP associations were negatively correlated with the S-A axis in SCZ, ASD, and PTSD (Fig. 3c,d), indicating an enrichment in the association cortex. Our results suggest that the spatial distribution of gene dosage effects differs by diagnosis. Similar correlations were observed with other functional and anatomical gradients that are also aligned with the S-A axis (e.g., T1w/T2w ratio reflecting myelin content; Fig. S3)^52^.

### Association of pathways with diagnosis varies by cell type and gene dosage

Our initial findings demonstrate that there are divergent genetic influences between different diagnostic categories when we stratify genetic effects by gene dosage and brain region. These findings highlight a principle that is somewhat obvious in retrospect. The multidimensional nature of psychopathology demands a multidimensional data analytic approach.

**Fig. 4|:**
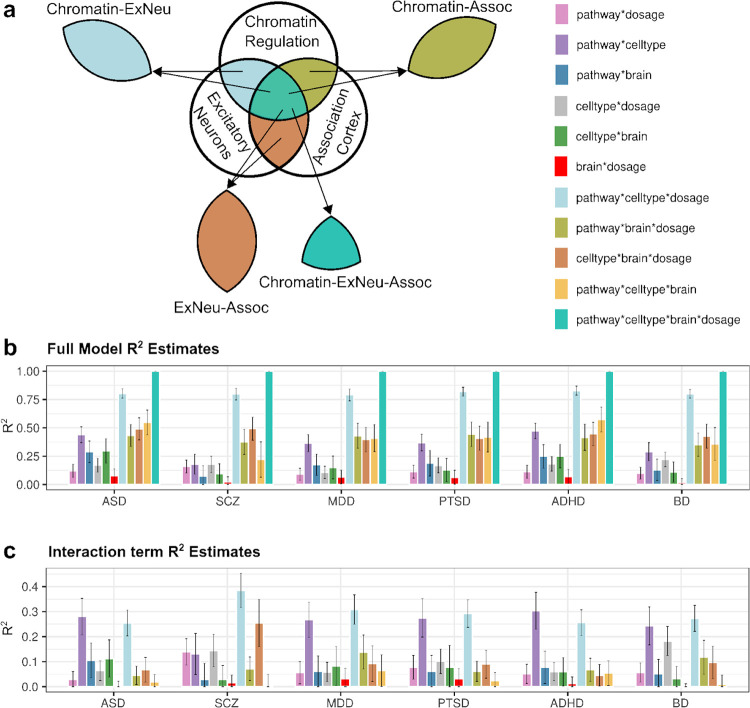
Associations of pathways with psychiatric traits vary by cell-type and gene dosage. (**a**) Schematic illustrating how gene sets were defined by intersecting pathway, cell type, and cortical region dimensions. Example intersections include Chromatin-ExNeu, Chromatin-Assoc, ExNeu-Assoc, and Chromatin-ExNeu-Assoc. (**b**) Full model R^2^ estimates showing the total variance in gene-set z-scores explained by main effects and interaction terms for each diagnosis. Models included pathway, cell type, brain region, dosage, and all combinations of two-way and three-way interactions. (**c**) R^2^ estimates for individual interaction terms, quantifying the contribution of each interaction to the explained variance. The pathway×celltype×dosage interaction consistently explains the largest proportion of variance across diagnoses, highlighting the importance of dosage-sensitive and cell-type-specific pathway effects (Tables S9–S10).

To characterize with more granularity how CNV effects are distributed in the brain, we investigated gene-dosage effects at the intersections of pathways, cell types, and brain regions. Pathway gene sets were intersected with cell types to create non-overlapping subsets (e.g., *Chromatin_ExNeu* and *Chromatin_InNeu*; Fig. 4a). Similarly, the transcriptome was divided into sensorimotor and association gene sets based on the correlations of individual genes with the S-A axis in the AHBA (76.29% of genes showed a nominally significant positive or negative correlation with PC1, Tables S6–S7). Pathways were intersected with these to create 2 region-specific subsets of each pathway (e.g., *Chromatin_Sensori*, *Chromatin_Assoc*). GSBA was then performed on two-way and three-way intersections of pathways (N = 19), cell types (N = 12), and brain regions (N = 2), including gene sets of size ≥30. Each gene set result was labeled with four factors: pathway, cell type, brain region, and dosage (Table S8).

We then evaluated which levels of biological organization best explain variation in gene-set effects within each diagnosis. We performed linear modeling on effect sizes of stratified gene-sets (z-scores) with different combinations of pathway, cell type, brain, and dosage as independent variables. For each diagnostic category, variance explained (R^2^) in summary statistics was calculated for main effects and interactions of these factors. Of all 2-way combinations, pathway and cell type explained the greatest variance (35.3% on average across diagnoses, Fig. 4b; Table S9). A full model that further stratified gene sets by dosage explained a majority of the variance (80.1% on average). The pathway×celltype×dosage interaction consistently explained the largest proportion of variance (Fig. 4c; Table S9), explaining half of the effect of the full model. This result highlights the importance of cell-type-specific and dose-dependent pathway effects across psychiatric conditions. Model fits improved by 7–20% when the brain region was included in the models (Fig. 4b,c; Tables S9–S10), suggesting that spatial variation in pathway expression also explains a proportion of variance.

### Diagnostic categories are differentiated based on gene-dosage effects in pathways by cell type and brain region

**Fig. 5|:**
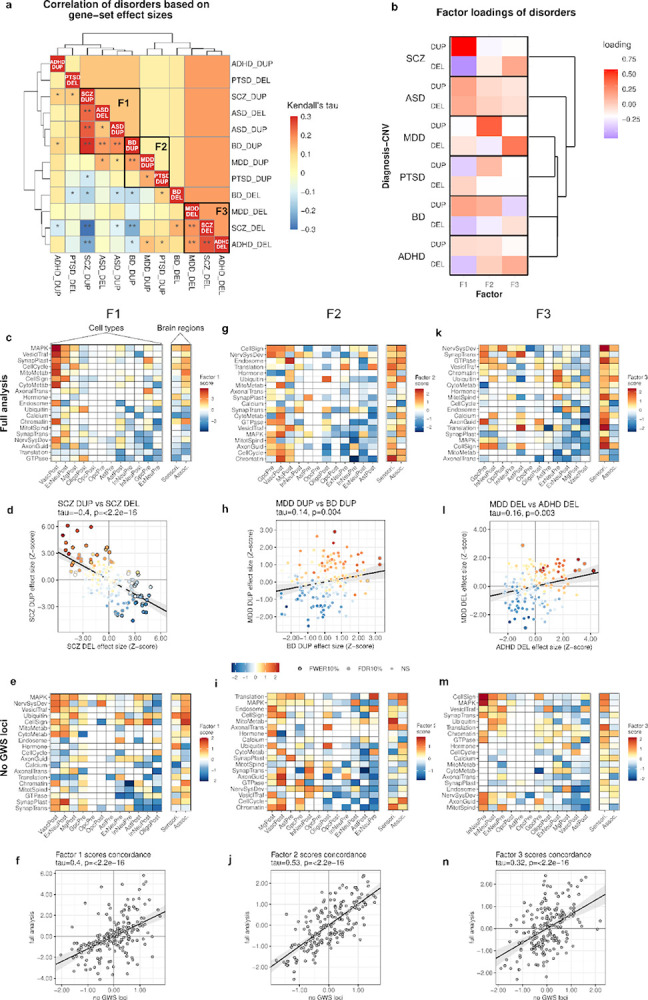
Differentiation of diagnostic categories based on gene-dosage effects in pathways by cell type and brain region. (**a**) Genetic correlations between diagnostic categories when each diagnosis-dosage combination is treated as an independent component, see also Table S11, *p<0.05) **q<0.05). Diagnosis-dosages with factor loadings >0.25 were grouped and labeled to highlight psychiatric traits contributing to F1, F2 and F3. (**b**): Factor loadings of DEL and DUP for disorders reveal a distinct profile for each diagnostic category. (**c, g, k**) Gene set-factor scores for the three factors, cell types and pathways were ordered using a simple sign-based bi-clustering algorithm (see methods) (Table S14). (**d**,**h**,**i**) Factor scores are representative of dose-dependent effects of genes. Scatterplots of gene set effect sizes (z-score) are shown for the top 2 diagnosis-dosage groupings with highest absolute factor loadings for factor F1, F2, and F3, and factor score of each gene set is indicated using the same color scale as in panels c,g,k. Solid trend lines indicate significant correlation between the diagnosis-dosage pair. (**e,j,m**) Factor analysis of gene sets with genome-wide significant loci removed yielded results with highly concordant gene set factor scores (**e,f,i,j,m,n**; tau_F1=0.45, tau_F2=0.53, tau_F3=0.32, p<2.2e-16; Table S14), demonstrating that these patterns are not attributable to a select subset of major loci.

To elucidate where gene-dosage effects converge at the intersection of pathways, cell types, brain regions, and psychiatric traits, we performed exploratory factor analysis (EFA)^60^ of functional gene sets to identify latent factors that correspond to different gene-trait relationships. Genetic correlations of DEL and DUP associations across 6 diagnostic categories were estimated based on gene-set summary statistics (Fig. 5a; Table S11). Factor analysis of gene-set summary statistics was performed to extract latent dimensions of genetic effects, and a three-factor model was optimal (Fig. S4). Factor **F1** captured dose-dependent effects in SCZ and BD (DUP positive, DEL negative) and dose-aligned effects in ASD (DUP positive, DEL positive) in shared gene sets. **F2** captured DUP effects shared by mood disorders and PTSD. **F3** captured DEL effects shared by MDD, ADHD and SCZ. Importantly, genetic correlations between diagnostic categories show greater contrast when DEL and DUP results for each disorder were treated as independent components (Table S11) compared to when all gene set tests for DEL and DUP were aligned between disorders (Fig. S5g; Table S12). This result is consistent with diagnostic categories having involvement of common functional processes with sometimes opposing directionality. Loadings of DEL and DUP effects onto the 3 factors yields a unique profile for each diagnostic category (Fig. 5b; Table S13).

The factor scores of functional gene sets show the relationships of neural processes to these latent dimensions. After a sign-based bi-clustering of the matrix, a structured pattern shows dose-dependent effects on pathways within cell types. **F1** in particular captures distinct clusters that represent the mirror-opposite effects of DUP and DEL seen in SCZ and other diagnostic categories (Fig. 5b). **Positively scoring gene sets** (Fig. 5c, upper left quadrant), which correspond to DUP associations in SCZ (Fig. 5 d), were enriched for core regulatory processes (cell cycle, MAPK, chromatin) and metabolic pathways expressed in postnatal neurovascular cells (VascPost), excitatory neurons (ExNeuPost), and microglia (MgPost) (Fig. S6). **Negatively scoring gene sets** (Fig. 5c, lower right quadrant), which reflect DEL associations in SCZ (Fig. 5d), were enriched for calcium signaling, axon guidance, and translation pathways expressed in inhibitory neurons and glia. F1 Factor scores also reveal divergent effects on synaptic transmission by cell type, with DUP associations concentrated in excitatory neurons and DEL associations in inhibitory neurons, a pattern that is consistent with a shift in excitatory-inhibitory balance. To assess whether these patterns might be attributable to strong signals from a select subset of loci, we repeated GSBA (Table S8) and factor analysis (Fig. 5e) after removing 18 loci that reached genome-wide significance (GWS) in our companion study^40^. The results showed highly concordant genetic correlations (Fig. S5c), factor solution and factor loadings (Fig. S5f), and gene-set factor scores (Fig. 5f,i,l). Thus the three factors derived in Figure 5 are not driven by a select subset of loci, and appear to be generalizable to CNVs genome wide. Similar clusters of pathway-cell type associations were evident in F1 (Fig. 5e), with the exception of the glial precursor cell type (GpcPre)(Fig. 2d). Lastly, F1 showed modest enrichment of gene set factor scores in Association cortex, a result that is consistent with the inverse dose-response of DEL (Fig. 3 b)and DUP (Fig. 3d) effects along the S-A axis. Supplementary figures are provided that illustrate all gene-set associations (Fig S6A), the subsets that are captured by each of the latent factors (Fig. S6B), and functional terms that are enriched within each factor (Fig. S6C).

The orthogonal **F2** factor showed divergent positive (associated with cases) and negative (associated with controls) effects in developmental signaling (cell-cycle, MAPK, GTPase signaling) pathways in non-neuronal and neuronal cell types, respectively (Fig. 5g,i). **Positively-scoring gene sets**, which correspond to positive DUP associations in mood disorders (Fig. 5h, Fig. S6b), include nervous system development and metabolic pathways concentrated in microglia (MgPost), and neurovascular cells (VascPost). **Negatively-scoring gene sets** correspond to negative DUP associations in similar pathways in neuroectodermal lineages (ExNeuPre, ExNeuPost, InNeuPre, AstPost; Fig. 5f,g). The patterns in F2 suggest that DUP effects in mood disorders are concentrated in core regulatory processes in non-neuronal cell types, while DUP effects in core regulatory pathways may be tolerated (or protective) in neurons with respect to diagnoses of MDD and BD. Thus, DUP effects on regulatory pathways in postnatal excitatory neurons (e.g. GTPase_ExNeuPost, CellCycle_ExNeuPost, Chromatin_ExNeupost) are a point of divergence between F1 and F2 that represents neural processes that are positively associated with SCZ and ASD and not associated with mood disorders (Fig. S6B–C).

**F3** was characterized by positive loadings of MDD-DEL and ADHD-DEL (Fig. 5b; Fig. S6b). **Positively-scoring gene sets** consisted of DEL effects in Cell-signaling and neurotransmission (SynapTrans, VesiclTraff) in inhibitory neurons (InNeuPre, InNeuPost). **Negatively-scoring gene sets** were broadly distributed across regulatory and metabolic pathways in microglia and neurovascular cells. Notably, nearly all (18/19) canonical pathways showed strong positive F3 factor scores in the sensorimotor cortex (Fig. 5i,k), consistent with the positive correlation of MDD-DEL and ADHD-DEL with the S-A axis in Figure 3a–b. Thus, F3 captures differential DEL effects in synaptic and regulatory pathways that vary along the S-A axis and in cell-type populations that align with this cortical expression gradient, such as inhibitory interneurons^55^.

### High-impact CNVs have a variety of cell-type specific gene-dosage effects

For CNV loci with the largest effect sizes on psychiatric traits, including reciprocal CNVs at 16p11.2, and 22q11.2^40^, clinical phenotypes are likely driven by the combined effects of multiple genes within each region^39,61–63^. Results from this study further suggest that a CNV may exert its influence through distinct pathway effects in multiple cell types.

**Fig. 6|:**
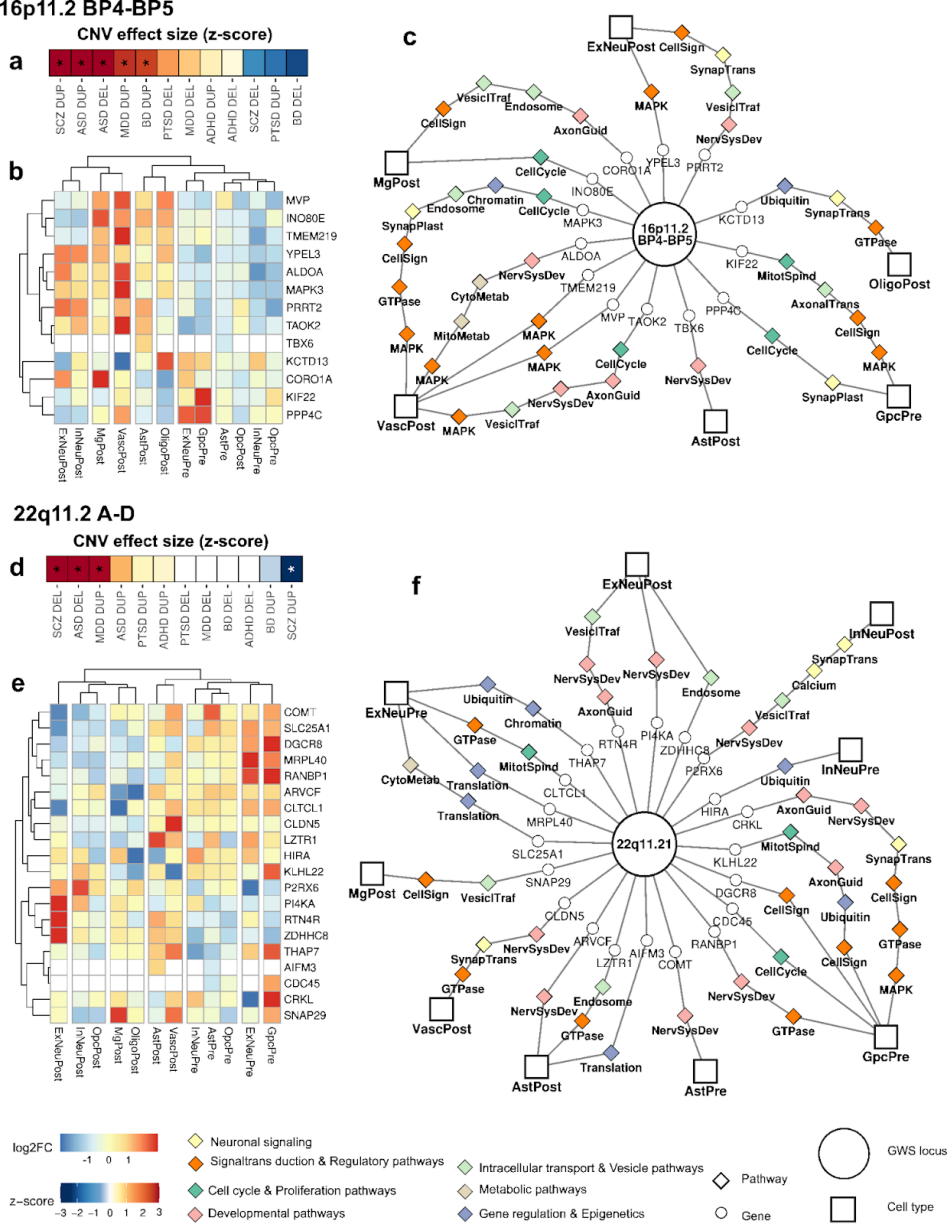
Cell-type specific expression of genes within major CNV loci 16p11.2 BP4-BP5 and 22q11.2 A-D suggests that the functional influence of a CNV in the brain may be driven by distinct pathway effects across a variety of cell types. CNV associations displayed in (**a**) and (**d**) were obtained from Shanta et al.^40^ Colors indicate the association direction and effect size (z-score), and asterisks indicate FDR<10% results. (**b**) and (**e**) heatmaps show log2 fold-change of cell type expression of the genes within each locus. The colors indicate the differential expression level. CNV-gene-gene-set networks in (**c**) and (**f**) display the CNV genes and their participation in the pathway-cell-type stratified gene sets. Shapes represent different entities of the network where the big circle in the middle is a GWS locus, peripheral circles are genes in the locus. A gene may be linked to one or more pathways (diamond) and at the end of the pathway, a cell type (square) is connected to indicate the gene membership of one or more stratified pathways of the same cell type. The color of diamond nodes indicates the group of pathways.

Duplication of 16p11.2 BP4-BP5 confers significant susceptibility to SCZ and BD, and Deletion is associated with ASD (Fig. 6a), consistent with some hallmarks of F1. Single-cell expression datasets^44^ confirm that expression of genes within the locus differs significantly by cell type (Fig. 6b), A network was constructed representing cell-type expression of CNV genes and pathways (Fig. 6c), highlighting several pathway-cell type effects that are consistent with positively-scoring gene sets on factor F1 including several genes tied to regulatory pathways in neurovascular cells (MAPK3, ALDOA, MVP, TMEM219, TAOK2) and microglia (*CORO1A*, *INO80E*) as well as MAPK signaling and synaptic plasticity in postnatal excitatory neurons (YPEL3 PRRT2).

The 22q11.2 A-D locus has mirror positive and negative effects of DEL and DUP respectively on SCZ susceptibility (Fig. 6d), which is also a hallmark of F1. Pathway-cell type effects in 22q11.2 are consistent with negatively-scoring gene sets on F1, including chromatin, translation and GTPase signaling in fetal excitatory neurons (SLC25A1, MRPL40, CLTCL1, THAP7), axon guidance and endosome recycling in postnatal excitatory neurons (RTN4R, POI4KA, ZDHHC8) and calcium signaling in postnatal inhibitory neurons (P2RX6)(Fig. 6e,f). As mentioned previously, gene set effects listed here, persist after removing all genome-wide significant loci. Thus, the functional gene sets enriched within major CNV loci generalize to gene-dosage effects in the rest of the genome.

## Discussion

We present an integrative framework for characterizing the functional convergence and divergence of rare genetic influences on mental health traits. Using a statistical genetic approach, gene set burden analysis (GSBA)^5^, we analyze the association of aggregate rare CNV burden in functional gene sets with diagnostic categories. A key element was to apply a multidimensional approach that quantified divergent effects of DEL and DUP in gene sets that represent the intersections of molecular pathways, neural cell types and cortical regions. This approach yields key insights into the neural basis of psychopathology. We demonstrate that, while major diagnostic categories converge on common molecular pathways, they diverge in the cellular context, spatial distribution, and directionality of genetic effects.

Gene-set burden tests identified 19 neurodevelopmental pathways, highly overlapping between ASD and SCZ, that were consistent with prior CNV^3,5^, WES^17,18^, and GWAS^15,64^ studies. These included pathways involved in neuronal signaling, GTPase and receptor mediated cell signaling, chromatin, translation, and metabolism. Cell-type associations included fetal excitatory neurons in ASD; excitatory neurons and oligodendrocytes in BD; and postnatal excitatory neurons, microglia, and neurovascular cells in SCZ. The involvement of neurovascular gene sets is notable given prior links of SCZ^65–67^, BD^68^ and ASD^32,69^ to cardiovascular disease. However, comparing lists of pathways and cell types does not reveal clear relationships between neural functions and diagnostic categories.

A key insight, originating from our companion paper^40^, is the dose-dependent effect of genes in SCZ and other diagnostic categories, evident by the inverse correlation of effect sizes for reciprocal DEL and DUP of the same genes. Stratification of pathway associations by gene dosage showed that pathway associations, particularly in SCZ, differ by dosage. SCZ-DUP effects were concentrated in core regulatory pathways and DEL effects in neuronal signaling.

In addition, incorporating spatial patterns of gene expression into GSBA revealed differential genetic effects across brain regions. In several diagnostic categories, the spatial distribution of gene dosage effects aligned with the S-A axis, a cortical gene expression gradient, extending from transmodal association areas (frontal, temporal cortex) to sensorimotor regions (visual, auditory cortex), and spatial distributions differed by diagnostic category, with DEL effects in MDD, ADHD and SCZ enriched in sensorimotor cortex, while DEL effects BD and DUP effects in ASD, PTSD and SCZ were enriched in association cortex.

These findings highlight how stratification of genetic effects by context and gene dosage allow for the differentiation of diagnostic categories. To determine where genetic effects converge and diverge at multiple levels, we investigated gene-dosage effects in the interactions of pathways, cell types and cortical regions. Mixed-effects modeling demonstrated that associations of gene sets captured the largest share of variance when pathways were stratified by cell type, and dosage. Spatial information also contributed a modest additive effect representing differential genetic effects along the S-A axis, as observed for MDD-DEL and ADHD-DEL (Fig. 3).

Factor analysis revealed three latent dimensions of gene-dosage effects (F1, F2, F3) that capture shared and distinct genetic architectures across diagnoses. A major factor **F1** captured a set of neural processes that have a dose-dependent relationship to SCZ (DUP positive, DEL negative) and dose-aligned relationship to ASD (DUP positive, DEL positive), with *distinct pathway-cell type combinations at opposing ends of the dose-response curve*. SCZ-DUP associations in cell-signaling (MAPK, cell-cycle) and metabolic pathways were concentrated in postnatal excitatory neurons and neurovascular cells. SCZ-DEL associations in neuronal signaling (synaptic, calcium) were concentrated in inhibitory interneurons, consistent with an imbalance of excitation and inhibition^70^. Dose-dependent effects in SCZ also correlated with the S-A axis (Fig. 3) with DUP effects aligned to the association cortex and DEL effects in sensorimotor regions. This pattern suggests that one major dimension of psychosis consists of negative effects on inhibitory activity (disinhibition) in sensory processing and positive dysregulation of excitatory processes in frontal/temporal regions. Thus, our genetic findings could inform studies of neurophysiology in schizophrenia^71,72^. Notably, ASD contrasts with SCZ in the directionality of effects in F1. In contrast to the dose-dependent effects in SCZ, In ASD, opposing effects of DUP and DEL are concentrated within the same neural processes. This fact could reflect distinct linear and non-linear dose responses for the cognitive traits underlying psychosis and social behavior respectively.

Additional factors captured orthogonal neural processes associated with mood disorders and ADHD. **F2** implicated cell-type specific effects in mood disorders consisting of divergent positive and negative effects on cell-signaling between non-neuronal and neuronal cells respectively, the latter being a point of divergence from SCZ and ASD. These findings represent a possible genetic basis for differences in the densities of neurons and glia that have been reported in postmortem studies of BD and MDD^26^. **F3** reflected differential DEL effects along the S-A axis capturing broad sensorimotor enrichment in ADHD and MDD consisting of synaptic and regulatory pathways in cell-type populations that align with this cortical gene expression gradient, such as inhibitory interneurons^55^.

We also show that specific high-impact CNVs are enriched for combinations of cell-type-specific genes involved in pathways consistent with our broader findings. 16p11.2 BP4-BP5^4^ represents a genomic region that is enriched for multiple functional gene sets at the positive end of factor F1 (cell signaling pathways in ExNeuPost and VascPost). Conversely 22q11.2 A-D^73^ is enriched for functional gene sets at the negative end of F1, such as regulatory pathways in ExNeuPre and calcium signaling InNeuPost. These results suggests that the large effects of an individual CNV may result from the combined impact of genes acting across multiple neural processes. Thus, 16p11.2 and 22q11.2 CNVs are monogenetic conditions that could serve as models for the dose-dependent effects of the major factor F1. High-risk CNVs, such as these represent patient groups that can be recruited for deep phenotypic characterization^74^ and parallel functional characterization of neural processes in brain organoid models^75,76^. Thus the findings from this study can be directly applied in clinical and translational studies of CNVs.

Our results provide a genetic basis for previous findings from other NIH-funded collaborations such as the PsychEncode consortium. Consistent with findings from Gandal et al., functional analysis of CNVs shows that core molecular pathways are shared by multiple diagnostic categories, such as ASD, SCZ, BD and MDD including synaptic transmission and neuronal signaling pathways^19^ and there are divergent effects in neuronal and non-neuronal cell types^20^. Considering just one level of biological organization at a time, such as pathways, the patterns that emerge from PsychEncode, GWAS, WES and CNVs are dominated broadly by “functional convergence” that seemingly spans all diagnostic boundaries. However, when genomic approaches take into consideration the joint influences of cell types, spatial distribution and directionality (dosage) of the pathway effects, distinct mechanisms emerge that underlie different dimensions of psychopathology.

## Methods

### 1. Participants and CNV data

The CNV subgroup of the Psychiatric Genomics Consortium (PGC) works in collaboration with principal investigators from many labs to obtain large sample sizes of microarray data and analyze them using a centralized pipeline. We acquired microarray intensity files from GWAS for a total of 574,965 samples that included data from cases and controls for 6 diagnostic categories (Table S1 in our companion paper^40^). These samples were genotyped on 25 platforms across 4 genome builds. Data from Illumina was collected as either raw intensity data (IDAT) files or final report files while data from Affymetrix was collected as CEL files. To harmonize data, probes for newly acquired datasets were lifted over to GRCH38 for CNV calling while previously called CNVs were lifted over to GRCH38. Samples were genotyped on either Illumina or Affymetrix array.

For samples that were provided as IDAT files, the Illumina command line version of Genome Studio was used in conjunction with platform-specific manifest and cluster files to produce genotype call (GTC) files. Relevant features were extracted from GTC files to obtain final report files with probes, genotypes, Log R Ratio (LRR), and B Allele Frequency (BAF) for each sample. For samples that were not mapped to GRCH38, probe genome positions were converted to hg38 using the LiftOver tool. Samples within each platform were grouped into batches by plate. For Illumina/PsychChip arrays, CNVs were called using two methods: PennCNV and iPattern. For Affy6 arrays, CNVs were called using four methods: PennCNV, iPattern, CScore, and Birdsuite. For Affy5 and Affy500K arrays, CNVs were called using two methods: PennCNV and Birdsuite. For Axiom arrays, CNVs were called using two methods: PennCNV and QuantiSNP. The consensus of CNV calls from multiple callers was created by merging CNVs at the sample level and retaining CNVs that were called by at least 2 methods.

#### 1.1 Sample QC

Quality control (QC) was performed first at the sample level, and conducted independently for each microarray platform, according to methods from our previous CNV GWAS of schizophrenia (Marshall et al. 2017^5^). For Illumina arrays, LRR standard deviation, BAF standard deviation, and GC waviness factor were extracted from PennCNV log files. Samples were retained if each of the measures were within 3 SD of the median. Affymetrix arrays used MAPD and waviness-sd parameters from affy power tools. Samples were further evaluated based on the number and total length of autosomal CNVs detected, and were retained if these values did not exceed 3 SD of the mean. The proportion of the chromosome that was tagged as a CNV was calculated and samples were excluded if >10% of the chromosome was marked as a CNV region to filter possible aneuploidies.

#### 1.2 CNV QC

Large CNVs that were fragmented were merged. CNVs <10kb in length or containing <10 probes were excluded. CNV calls were removed if they spanned the centromere or telomere (100kb from end of chromosome) or had >50% overlap with segmental duplications, immunoglobulin, or T cell receptor (recurrent CNVs were processed without segmental duplications, immunoglobulin, and T cell receptor filters). The call set was restricted to rare CNVs with ≤10% frequency within-platform or across all platforms.

### 2. Ancestry Principal Components and Ancestry Partitioning

We extracted a subset of SNPs with < 1% missingness across all platforms (12,185 SNPs) and performed a principal component analysis using the flashPCA software^78^. In order to genetically infer the ancestry of each individual, we used the SNPweights software^79^ on the same subset of SNPs to calculate % ancestry based on a reference panel containing 6 different populations (751 EUR, 687 EAS, 630 SAS, 568 AFR, 41 AMR, 22 OCE). Samples were categorized into 5 large homogeneous groupings based on the following criteria used in a previous study^80^ 39 (Table S2, Fig S1): EUR: subjects with EUR ≥ 90%, AFR/AFAM: subjects with EUR < 90% & AFR ≥ 5% & EAS/SAS/AMR/OCE < 5%, ASN/ASAM: subjects with EUR < 90% & (EAS ≥ 5% or SAS ≥ 5%) & AFR/AMR/OCE < 5%, LAT: subjects with EUR < 90% & AMR ≥ 5% & EAS/SAS/AFR/OCE < 5% or EUR < 90% & AMR ≥ 60% & EAS < 20% & SAS < 15% & AFR/OCE < 5%, MIX: Uncategorized subjects.

### 3. Gene QC

To avoid having false positive findings arising due to a platform or dataset biases, we performed an extra filtering step of the genes being included in the gene set analysis. For each gene, separately for DELs and DUPs, CNV frequency was calculated per platform and dataset. Given the reduced penetration of the most recurrent CNVs, the incident frequency of such CNVs can be higher than that of disease prevalence. In particular, 15q11.2 DEL (major risk locus for ASD and SCZ) has been reported to have an incident rate between 0.57–1.27 %^81^, thus, using an inclusive frequency threshold, wWe then limited the CNVs to those with frequency lower than 2% across platforms and datasets. In addition, we calculated weight deviance score (WDS) of CNV frequency per platform/dataset and used that to derive a platform/dataset specificity index (SI). Specifically, for each gene, CNV frequency (C_i_) for a particular platform/dataset was compared to the expected CNV frequency (E_i_) estimated from across platforms/datasets as shown in Eq.1.

(Eq.1) $${\text{E}}_{\text{i}}={\text{N}}_{\text{i}}*{\text{C}}_{\text{all}}/{\text{N}}_{\text{all}}$$

where for a particular platform/data i, E_i_ is the expected CNV frequency, N_i_ is the sample size, C_all_ is the CNV frequency in the entire dataset, and N_all_ is the entire dataset sample size.


(Eq.2) $${\text{WDS}}_{\text{i}}=\left({\text{C}}_{\text{i}}\text{-}{\text{E}}_{\text{i}}\right)/\text{sqrt}\left({\text{E}}_{\text{i}}*{\text{N}}_{\text{i}}\right)$$

Then WDS_i_ was calculated as Eq. 2. With the max WDS across platforms/datasets representing the specificity index. We removed genes having dataset_SI ≥ 0.2 and platform_SI > 0.6 from subsequent analyses.

### 4. Gene set data

#### 4.1 Cortical regions

To generate gene sets for different cortical regions of the human brain, we acquired gene expression data in the brain from Allen Human Brain Atlas (AHBA; https://human.brain-map.org/static/download)^46^, multimodal brain parcellation from Glasser’s brain regions^45^. Using the Abagen toolbox (version 0.1.3; https://github.com/rmarkello/abagen)^82^, we mapped brain parcels and gene expression data, and then performed gene expression normalization and scaling. Specifically, a robust sigmoid function was used to normalize the expression data across genes to address inter-sample variation, while min-max normalization was applied after to scale the gene expression across tissue samples. Using the left hemisphere, we defined 180 regions from Glasser’s brain regions^83^. To generate the gene sets, the region-mean expression levels of each gene were z-transformed across the regions. Genes were then assigned to cortical region(s) when their z-score>1. The median gene set size was 4,429 genes (see Table S1). To visualize cortical region results, we used ggseg v1.6.5^84^ and ggsegGlasser R libraries for Glasser’s brain regions.

#### 4.2 Cell types

We obtained single-cell RNA-seq data from Velmeshev et al., 2023^44^, which contains the data >700,000 nuclei covering both prenatal and postnatal development periods and 8 defined cell type clusters. The 8 defined cell type clusters were 1. Oligodendrocyte precursor cells (OPC), 2. Vascular cells (Vasc), 3. Excitatory neurons (ExNeu), 4. Oligodendrocytes (Oligo), 5. Interneurons (InNeu), 6. Microglia (Mg), 7. Astrocytes (ASst), and 8. Glial progenitors (Gpc). Using the cluster result from the original study, we redefined the cluster by taking into account the developmental period of the cell. Doing so, we obtained 12 cell type clusters; 1. postnatal Opc, 2. postnatal Vasc, 3. postnatal ExNeu, 4. postnatal Oligo, 5. postnatal InNeu, 6. postnatal Mg, 7. postnatal Ast, 8. prenatal ExNeu, 9. prenatal Ast, 10. prenatal Opc, 11. prenatal InNeu, and 12. prenatal Gpc. We then generated cell type marker gene sets using FindAllMarkers() function from the Seurat package. Genes were assigned to a particular cell type cluster with the highest average log2 fold-change only when the corresponding p-value is < 0.05 (Table S1). The gene set size for cell types were smallest in prenatal OPC (181 genes), and largest in postnatal Mg (2,058 genes) with a median of 1,223 genes.

#### 4.3 Molecular pathways and pathway clusters defined using EnrichmentMap

We compiled gene sets from multiple databases including Gene Ontology^41^, KEGG pathways^42^ and Reactome^43^. We filtered the gene sets to include only those with size between 50 and 500 genes, excluding sets with broader definition (>500 genes) and those with low statistical power (<50 genes). In total, we acquired 2,453 gene sets. To reduce dependency between tests for multiple testing correction, we further exclude 758 more gene sets through a step-down approach. Specifically, for each gene set, we removed any smaller subset with substantial gene overlap (Jaccard’s index >0.75). The gene set sizes for molecular pathways range from 50 genes to 495 genes with a median of 145 genes.

To summarize the pathway associations, we applied the EnrichmentMap Cytoscape plugin^49^ on the top associated gene sets (BH-FDR<5%, with z-score>0) from all the conditions. There were 361, 106, 7, and 5 gene sets associated with SCZ, ASD, BD, and ADHD, respectively. By limiting to pathway clusters with at least 3 gene set members, this results in 19 pathway clusters. We then constructed new gene sets by merging all gene sets within each cluster for subsequent analyses.

### 5. Gene set burden analysis and sample-weighted meta analysis

Differences in genotyping platforms have been known to confound CNV detection given the variance in probe coverage. While the most common way to tackle platform bias in CNV data analysis is to model the effect as one of the covariates, however, the effect is not well controlled in a single regression model. In this study, we performed gene set burden analysis independently for different genotyping platforms and meta-analyzed the summary statistics derived from the individual platform analysis. Using ASD and SCZ as a preliminary experiments, in both conditions, we found a smaller genomic inflation factor or lambda (λ) value (Eq.3) in the meta-analysis result (λ_ASD_=1.78, λ_SCZ_=3.35) compared to the mega-analysis result (using platform as a covariate, λ_ASD_=1.82, λ_SCZ_=3.66).

(Eq.3) $$\lambda =\text{median}\left(\chi^{2}\right)/0.455$$

where χ^2^ is chi-square statistics, and 0.455 is the theoretical mean of chi-square distribution.

Specifically, we performed the gene set analysis on platforms where there are at least 50 cases and 50 controls. For each platform, a univariate analysis was conducted to compare the burden of genes in a gene set impacted by DELs or DUPs between cases and controls. The univariate analysis was done in one of two ways, either 1) a traditional case-control comparison for each individual condition, or 2) a family-based comparison. For the traditional case-control comparison, logistic regression was applied by regressing the number of genes in a gene set impacted by DELs or DUPs on the affection status (1 = affected, 0 = unaffected). Population structure (PC1–10), sex, and the number of genes outside the gene set impacted by DELs or DUPs were used as covariates to correct for any biases in the population, sex and total burden load. For the family-based comparison, we applied conditional logistic regression the same way logistic regression was applied, except that samples were matched by family ID. A likelihood ratio test was done to estimate p-value by comparing two regression models with and without the testing variable, in this case, a gene set burden.

A sample-weighted meta-analysis was applied to account for substantial differences in sample size between platforms. We derived the weight for each platform based on the effective sample size as shown in Eq.4.

(Eq.4) $${\text{Weight}}_{\text{i}}=\text{sqrt}\left(4/\left(1/{\text{Ncase}}_{\text{i}}+1/{\text{Nctrl}}_{\text{i}}\right)\right)$$

where Ncase_i_ is the number of cases in platform_i_, and Nctrl_i_ is the number of controls in platform_i_.

### 6. Gene burden analysis

We generated gene-level summary statistics by meta-analyzing the summary statistics from individual platform gene burden analysis. Similar to the gene set burden analysis, the gene burden analysis was done by either performing a logistic regression for case-control dataset, or conditional logistic regression for family-based dataset. We regressed the status of the CNV whether or not a sample has DELs or DUPs overlapping a particular gene on the affection status of the condition. Like gene set burden analysis, population structure (PC1–10), and sex were corrected in the analysis, with family ID being a random effect variable for conditional logistic regression. As multigenic CNVs might drive correlation between tests and that would affect multiple testing correction, genes were merged when the Jaccard index estimated from the proportion of CNVs commonly found between genes was >0.75. Since we only used the gene burden results to visualize findings from the main analysis, we did not report them in this study.

### 7. Correlation analysis of CNV association and Sensorimotor-Association axis and pathway-S-A-axis gene set stratification

We investigated how CNV associations distributed along the cortical gradient using the dominant brain transcriptomic variance data compiled in Dear et al^51^. This is the PC1 of AHBA transcriptomic profile^46^ projected on the Glasser parcellation^45^. The data was processed to exclude spatially inconsistent genes and, under sampling parcellations with a low number of donors (<6 donors). As a result, the final principal component analysis was performed on 134 parcellations and 7,937 genes. The CNV meta-analysis summary statistics of 134 Glasser parcellations was then compared with the PC1 AHBA using Spatial Permutation Inference (SPIN test^59^ with 10,000 permutations) with Kendall coefficient analysis.

To stratify gene set by the S-A axis, we first compute the Kendall coefficient of each gene against the PC1 AHBA. The gene expression matrix was preprocessed and obtained from Dear et al^51^ where it contains the data for 10,028 genes, of which 8,588 genes are a member of at least one gene set. This identified ~76% of the genes (n=6,552) to be correlated with the S-A axis at nominal significant level (p<0.05). We then stratified each gene set into 1) sensorimotor cortex set (tau>0, p<0.05), and 2) association cortex set (tau<0, p<0.05), leaving out other non-correlated genes from the subsequent analysis.

### 8. Genetic correlations based on gene-set summary statistics

We compared each pair of summary statistics (e.g., a pair of DEL and DUP summary statistics) 1) within the same condition to assess dosage sensitivity at the gene set level in each condition, and 2) between two conditions to assess gene set profile similarity between conditions. To do so, we performed a Kendall rank correlation analysis of the z-scores estimated from the meta-analysis of gene set burden results across individual platforms.

To examine correlations between cortical maps (e.g., CNV associations, transcriptomic gradient map, etc.), we applied a commonly used spatial Kendall’s correlation and assessed significance against a two-sided spatial autocorrelation-preserving null mode (SPIN test)^59^, accounting for high inter-regional correlations as a result of spatial smoothing. To reduce the influence of gene set size on the z-score and the estimated correlation, we regressed out the gene set size from the z-score and performed correlation analysis on the residuals.

Using stratified gene set summary statistics, we estimated genetic correlations in 2 ways: (1) First, was to treat each diagnosis-dosage combination as an independent component and to examine the correlations of each (12 × 12, Table S11). (2) Second we examined the correlation of all the gene-set effects combined by stacking DEL and DUP summary statistics and aligning them directly between diagnoses (Table S12). Genetic correlations of independent diagnosis-dosage combinations showed greater contrast between diagnostic categories (Fig. S5g).

### 9. Latent factor analysis

We performed a latent factor analysis on the two-way (pathway-cell-type, pathway-brain) and three-way (pathway-cell-type-brain) stratified gene set summary statistics to investigate the shared and convergent dosage effects amongst the 6 psychiatric conditions using psych R library. The number of factors was optimized using a scree plot (elbow plot) of PCA on the summary statistic where a 3-factor solution was chosen (Fig. S4).

We specified a 3-factor solution using the fa() function, which estimates factor loadings that describe how each diagnosis-dosage combination contributes to the latent factors. A heatmap of the factor loading matrix, styled similarly to the genomic SEM plots, was generated to visualize how diagnoses align with these latent dimensions. To relate individual pathway gene sets to the latent factors, we computed the factor score for each gene set as a product between their z-scores and the factor loadings across diagnosis-dosage combinations, producing a second heatmap that highlights which biological pathways align most strongly with each latent genetic factor. For this second heatmap, specifically for pathway-cell-type stratified gene sets, we performed a sign-based bi-clustering on the heatmap where pathways are in rows, and cell types are in columns. The pathways and cell types were ordered in a descending order based on their average factor scores. This resulted in two main clusters of groups of pathways and cell types for i) positive and ii) negative factor scores.

All 2 way and 3 way stratification were included in the mixed-effects model analysis (Fig. 4). A factor analysis of three-way stratified gene sets (Fig. S8) produced similar factor solutions, genetic correlations and factor scores as the results in Figure 5. However, overall signal was comparatively weak due to the sparsity of the counts in the 3-way stratification of the data and the sparsity of gene sets that could be included in the analysis (stratifying pathway, by celltype, brain and dosage resulted in >50% of gene sets meeting the minimum size of 30 genes, Fig. S7). Thus main results in Figure 5 include only the 2 way (pathway-cell type and pathway-brain) gene sets.

### 10. Linear model analysis investigating variance explained by different genetic factors

Using stratified gene set summary statistics, we evaluated which levels of biological organization best explain the variation in the gene-set effects within each diagnosis. We performed linear modeling on the effect sizes of the stratified gene-sets (z-scores) with different combinations of pathway, cell type, brain, and dosage as independent variables. For each diagnostic category, variance explained (*R*^*2*^) in summary statistics was calculated for the full model, the main effects, and the interactions of these factors. For example, suppose we would like to test for the effect of a cell type variable and its interaction term. Let *m0* be the full model of all three variables (e.g., logit(y) ~ pathway*celltype*dosage), *m1* be the model without the interaction term with all three variables (e.g., logit(y) ~ pathway*celltype + celltype*dosage), *m2* be the additive model without the evaluating variable (e.g., *logit(y) ~ pathway + dosage*), and *m3* be the additive model with all three variables (e.g., *logit(y) ~ pathway + celltype + dosage*). The *R*^*2*^ of the full model is from m0, the *R*^*2*^ of the main effect is estimated as *R*^*2*^*m3*-*R*^*2*^*m2*, and the *R*^*2*^ of the interaction term is estimated as *R*^*2*^*m0*-*R*^*2*^*m1*. A likelihood ratio test was performed to estimate the level of significance for each comparison through the *anova()* function in R.

## Supplementary Material

Supplement 1

1

## Data Availability

A WDL workflow containing all steps of CNV calling, QC and CNV-GWAS and meta-analysis code is under construction and will be released on the PGC CNV Github in conjunction with this publication (https://github.com/orgs/psychiatric-genomics-consortium/teams/cnv). Analysis code for GSBA and downstream analyses (https://github.com/naibank/PGC_GSBA) Gene sets, see Table S1, Gene-set summary statistics, see Table S3. Raw genotype and intensity files are available on subset of the cohort PGC dbGAP datasets https://www.ncbi.nlm.nih.gov/projects/gap/cgi-bin/collection.cgi?study_id=phs001254.v1.p1 Simons Foundation Autism Research Initiative SFARI (SSC and SPARK) https://base.sfari.org/
