# Supplementary material for: Psychiatric disorders converge on common pathways but diverge in cellular context, spatial distribution, and directionality of genetic effects
Source: medRxiv. 2025 Jul 16:2025.07.11.25331381. Preprint. [Version 2] doi: 10.1101/2025.07.11.25331381 (PMC12338884; doi:10.1101/2025.07.11.25331381)
Supplement: 1 [file NIHPP2025.07.11.25331381V2-supplement-1.pdf]

medRxiv preprint doi: https://doi.org/10.1101/2025.07.11.25331381; this version posted July 16, 2025. The copyright holder for this preprint (which was not certified by peer review) is the author/funder, who has granted medRxiv a license to display the preprint in perpetuity.
It is made available under a CC-BY 4.0 International license .

Supplementary materials

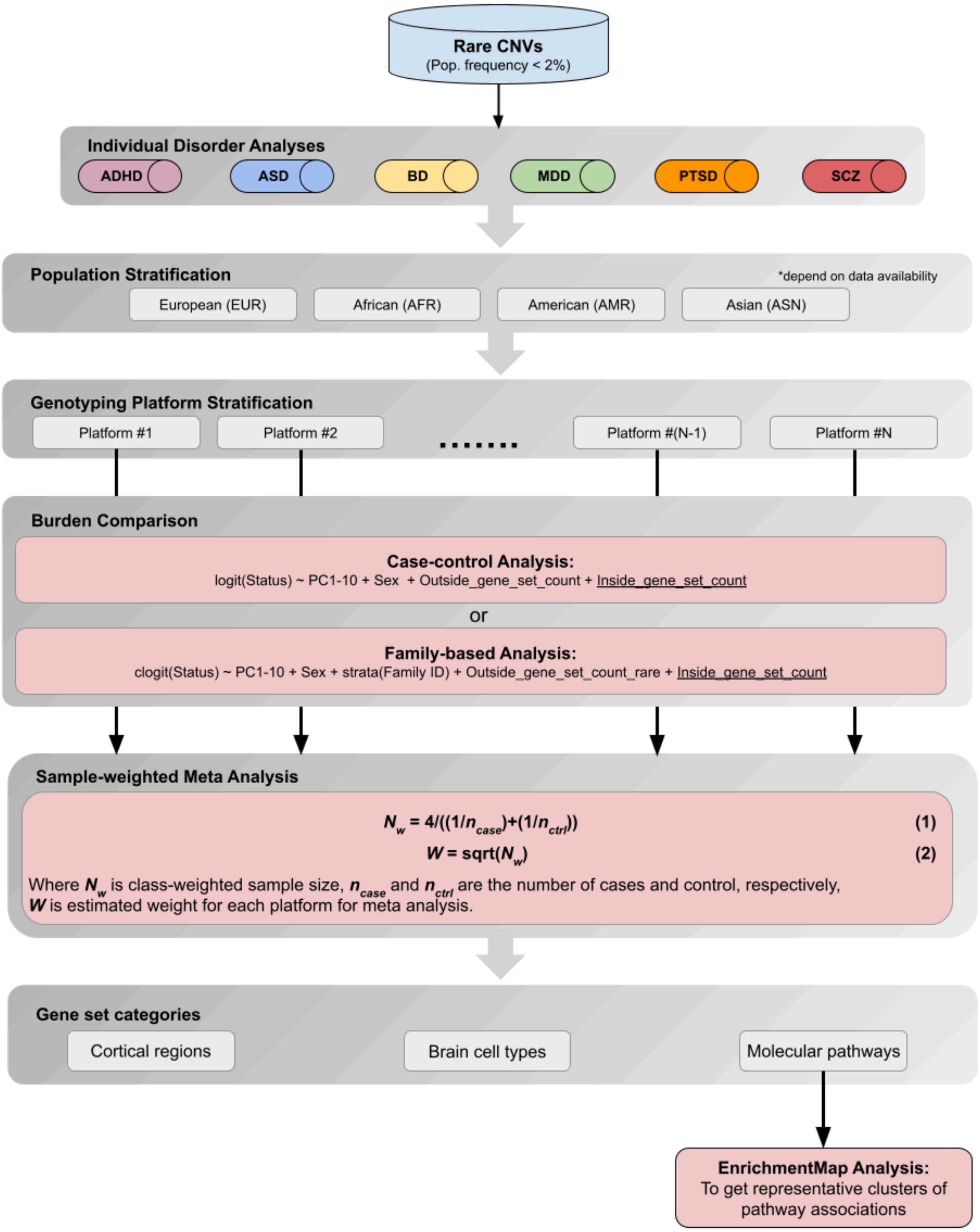

Fig. S1 | Gene set burden analysis (GSBA) workflow

A diagram showing the analytical procedure done for the gene set analysis of CNV data. First, CNVs were called and filtered down to rare CNVs at 2% frequency across platform and ancestry. Then, for each individual condition, to maximize the statistical power, we performed a cross-ancestry analysis, and also stratified the analysis by population groups; European (EUR), African (AFR), American (AMR), and Asian (ASN). For each stratified analysis, the gene-set burden comparison were done independently for each genotyping platform, then their summary statistics were meta-analyzed. For the burden comparison, we

medRxiv preprint doi: https://doi.org/10.1101/2025.07.11.25331381; this version posted July 16, 2025. The copyright holder for this preprint (which was not certified by peer review) is the author/funder, who has granted medRxiv a license to display the preprint in perpetuity.
It is made available under a CC-BY 4.0 International license .

either performed a logistic regression for case-control data, or a conditional logistic regression for family-based data where family ID was used as a strata. Meta-analysis was done using a sample-weighted procedure (Eq.3-4), as it has shown a better robustness compared to a standard-error-based procedure. For the result of molecular pathways, we further clustered them using EnrichmentMap to obtain representative pathway clusters.

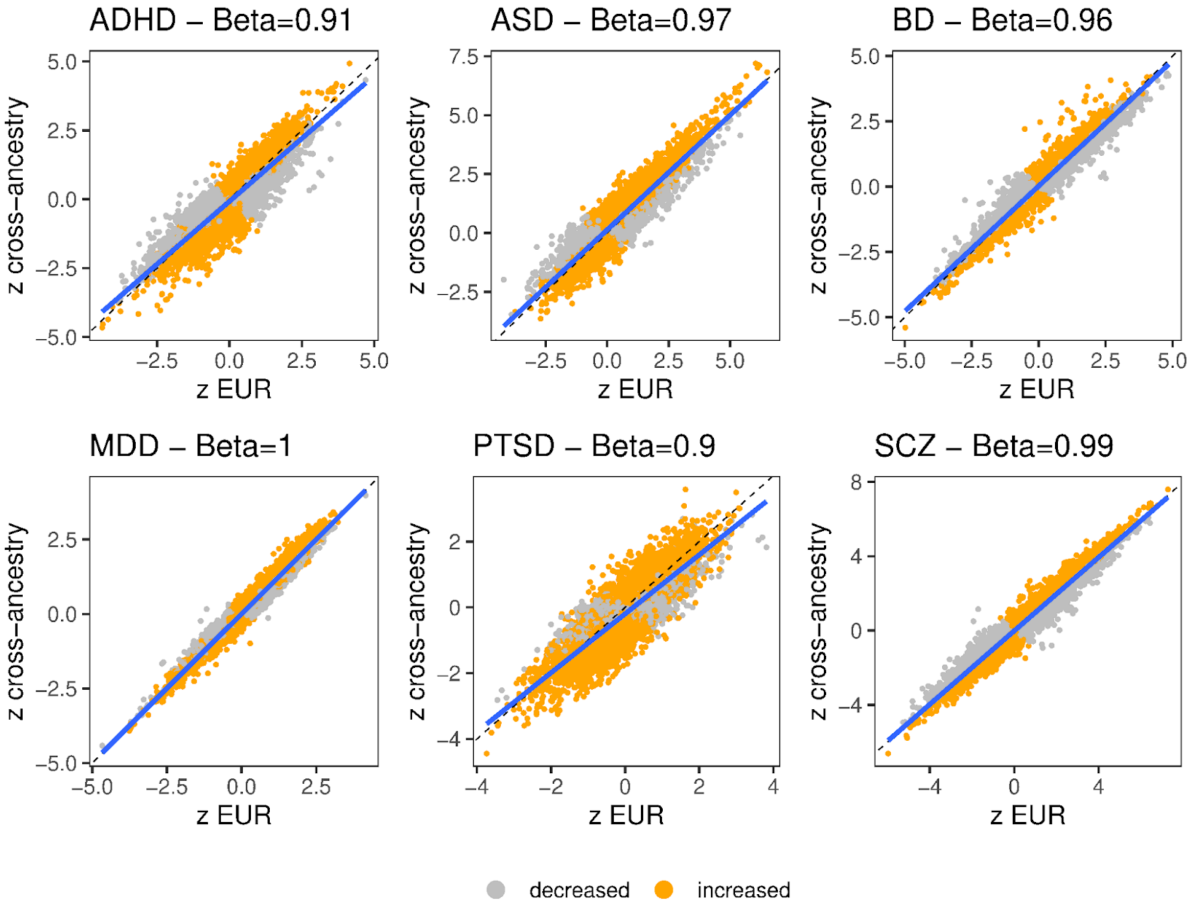

Fig. S2 | Comparing GSBA results between the full cohort and subjects of only European ancestry - scatter plots comparing summary statistics (z statistics from the sample-weighted meta analysis) between the analysis of European subset and the analysis of all ancestry. Beta coefficients estimated from linear model regressing z statistics from European analysis on the z statistics of cross-ancestry analysis.

medRxiv preprint doi: https://doi.org/10.1101/2025.07.11.25331381; this version posted July 16, 2025. The copyright holder for this preprint (which was not certified by peer review) is the author/funder, who has granted medRxiv a license to display the preprint in perpetuity.
It is made available under a CC-BY 4.0 International license .

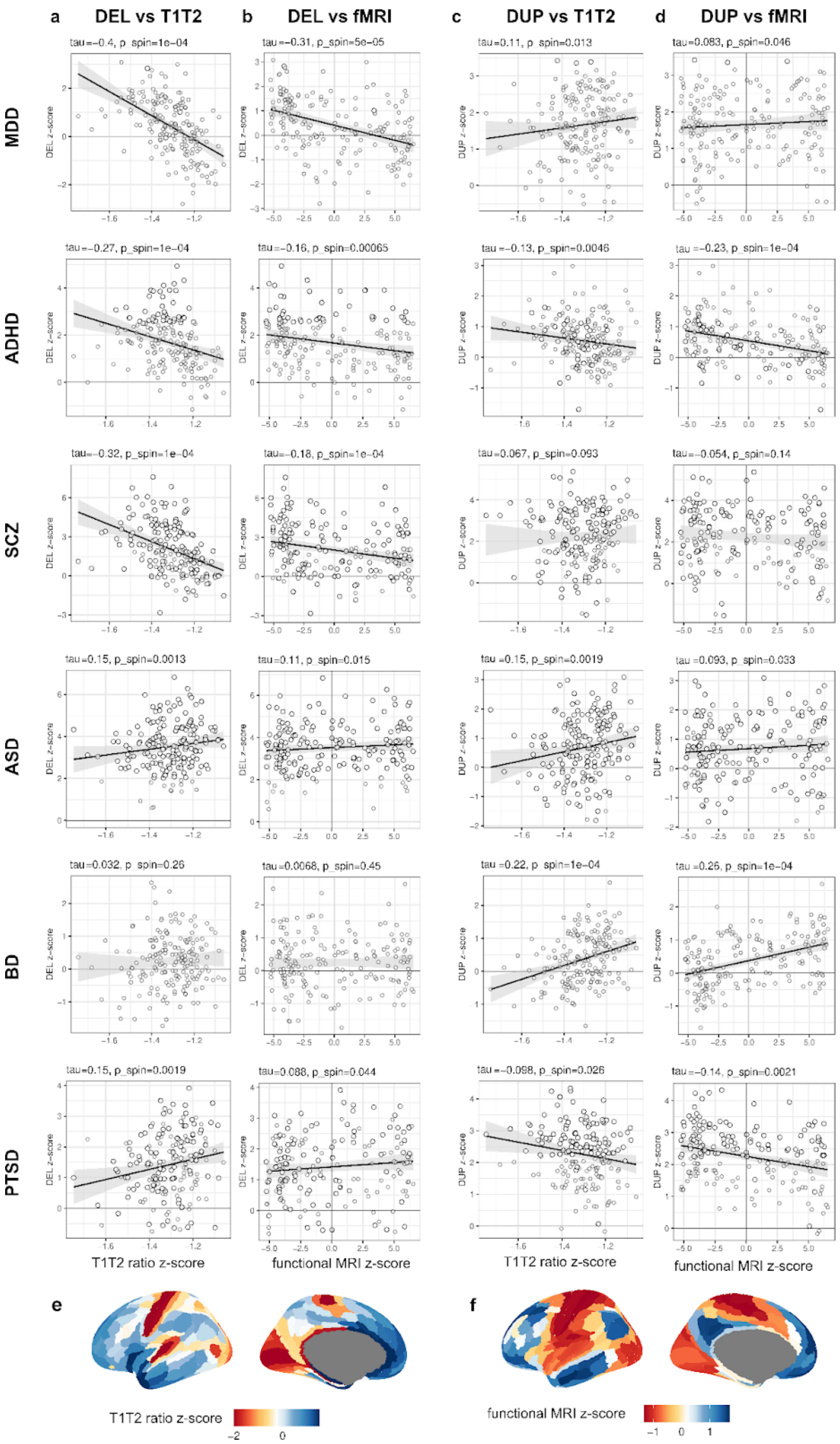

Fig. S3 | Correlation of cortical gene set effects with additional sensory–association cortical gradients. Scatter plots show the correlation between gene-set burden z-scores for DEL (**a,b**) and DUP (**c,d**) for two independent measures of cortical organization: the T1w/T2w ratio which reflects regional variation in intracortical myelination, and the principal gradient of resting-state fMRI. T1w/T2w and fMRI measures aligned to the Glasser grain maps were obtained from Markello et al. [52] , both of which parallel the S-A axis derived from transcriptional principal components(Fig. 3). Correlation of CNV effects with these gradients

medRxiv preprint doi: https://doi.org/10.1101/2025.07.11.25331381; this version posted July 16, 2025. The copyright holder for this preprint (which was not certified by peer review) is the author/funder, who has granted medRxiv a license to display the preprint in perpetuity.
It is made available under a CC-BY 4.0 International license .

supports the spatial specificity of gene-dosage associations across multiple cortical modalities. Together, these analyses highlight the convergent spatial patterning of CNV effects along major anatomical and functional cortical hierarchies. (**a**) DEL z-score and T1-T2 ratio, and (**b**) DEL z-score and fMRI. (**c**) DUP z-score and T1-T2 ratio, and (**d**) DUP z-score and fMRI. Solid trend lines indicate significant correlation where p_{SPIN}<0.05. Brain maps of T1-T2 ratio and fMRI are shown in (**e**) and (**f**) where colors indicate the z-score.

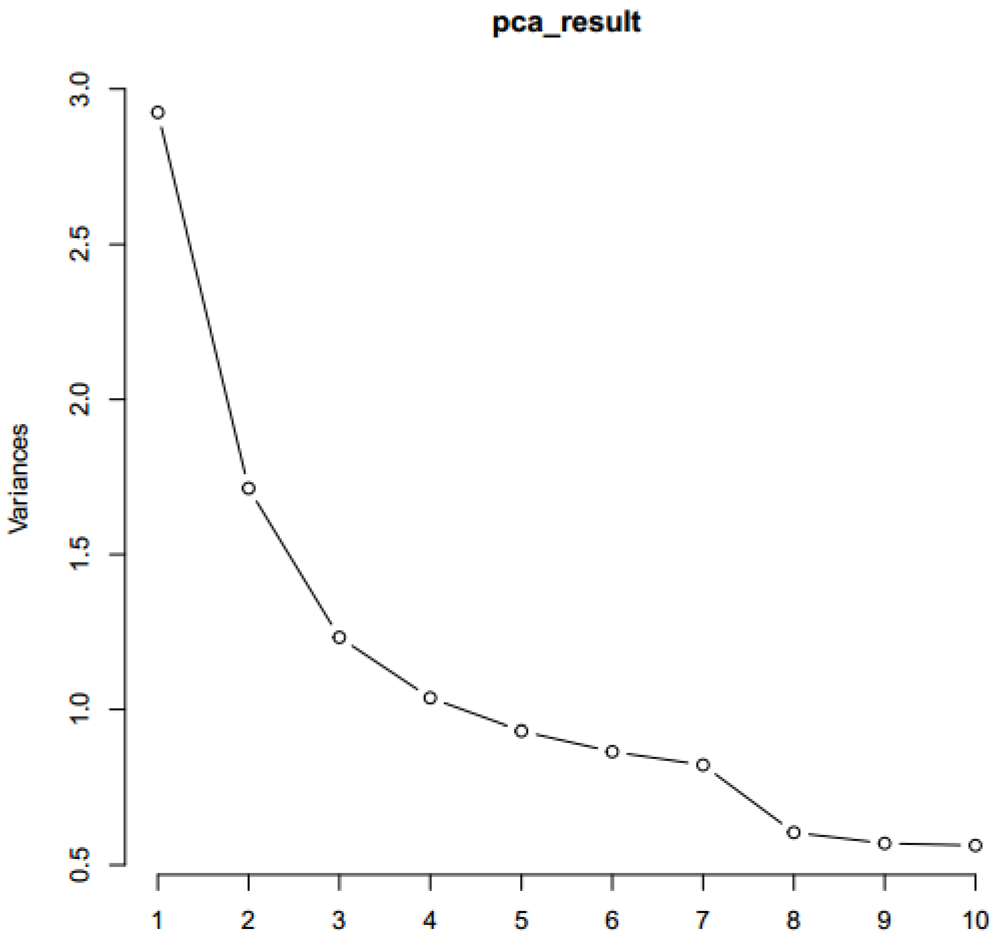

Fig. S4 | Using elbow plot (scree plot), we estimated an optimal number of factors to be 3 factors (variance drop threshold<5)

medRxiv preprint doi: https://doi.org/10.1101/2025.07.11.25331381; this version posted July 16, 2025. The copyright holder for this preprint (which was not certified by peer review) is the author/funder, who has granted medRxiv a license to display the preprint in perpetuity.
It is made available under a CC-BY 4.0 International license .

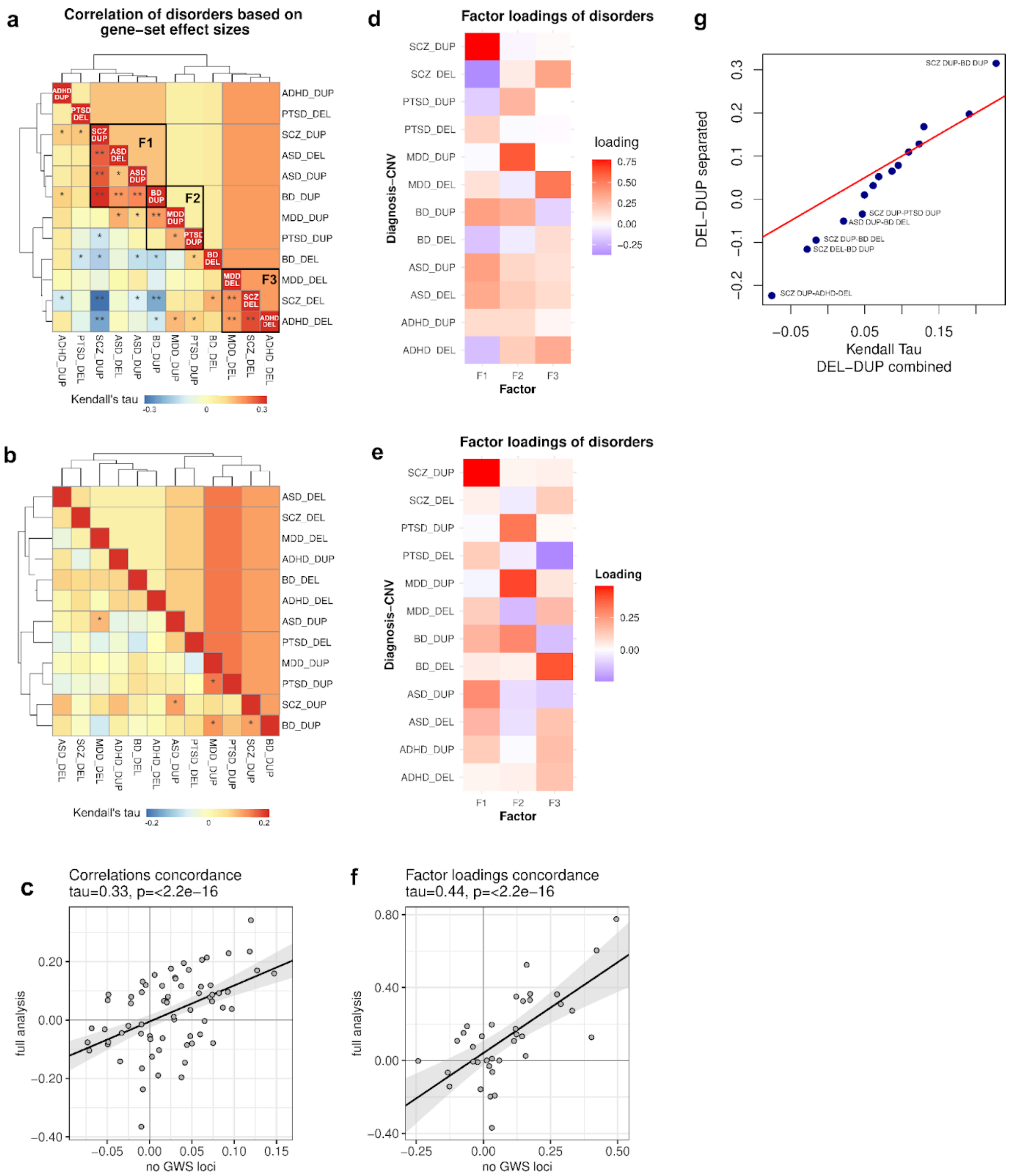

Fig. S5 | Factor analysis on two-way pathway stratification summary statistics without (by cell type, and by brain region) genome-wide significant (GWS) loci included in the analysis. Genetic correlations between diagnosis-dosage from (**a**) the full analysis and (**b**) the analysis without GWS loci. Single asterisks (*) indicate nominal significance (p<0.05), while double asterisks indicate significance after multiple testing correction (q<0.05), and a factor loading threshold of > 0.25 was applied to determine factor members. (**c**) Correlation of genetic correlation calculated from full analysis and no GWS loci analysis. Factor loadings of diagnoses reveal distinct signatures of diagnostic categories from (**d**) the full analysis and (**e**) no GWS loci analysis. (**f**) Correlation of factor loadings from full analysis and no GWS loci

medRxiv preprint doi: https://doi.org/10.1101/2025.07.11.25331381; this version posted July 16, 2025. The copyright holder for this preprint (which was not certified by peer review) is the author/funder, who has granted medRxiv a license to display the preprint in perpetuity.
It is made available under a CC-BY 4.0 International license .

analysis. For (**c**) and (**f**) scatterplots, solid trend lines indicate significant correlation. Kendall's Tau and corresponding p-value are reported in the title of the scatterplot. (**g**) QQ-plot comparing the distributions of correlation coefficients (Kendall's Tau) when DEL and DUP effects in each diagnosis are treated as separate components (y-axis, Table S11) vs when the full sum stats of DEL and DUP are aligned between diagnoses (x-axis, Table S12) . The negative tail of the y-axis distribution on the QQ plot was weakly skewed, suggesting that the distribution was enriched for effects that diverge between diagnoses.

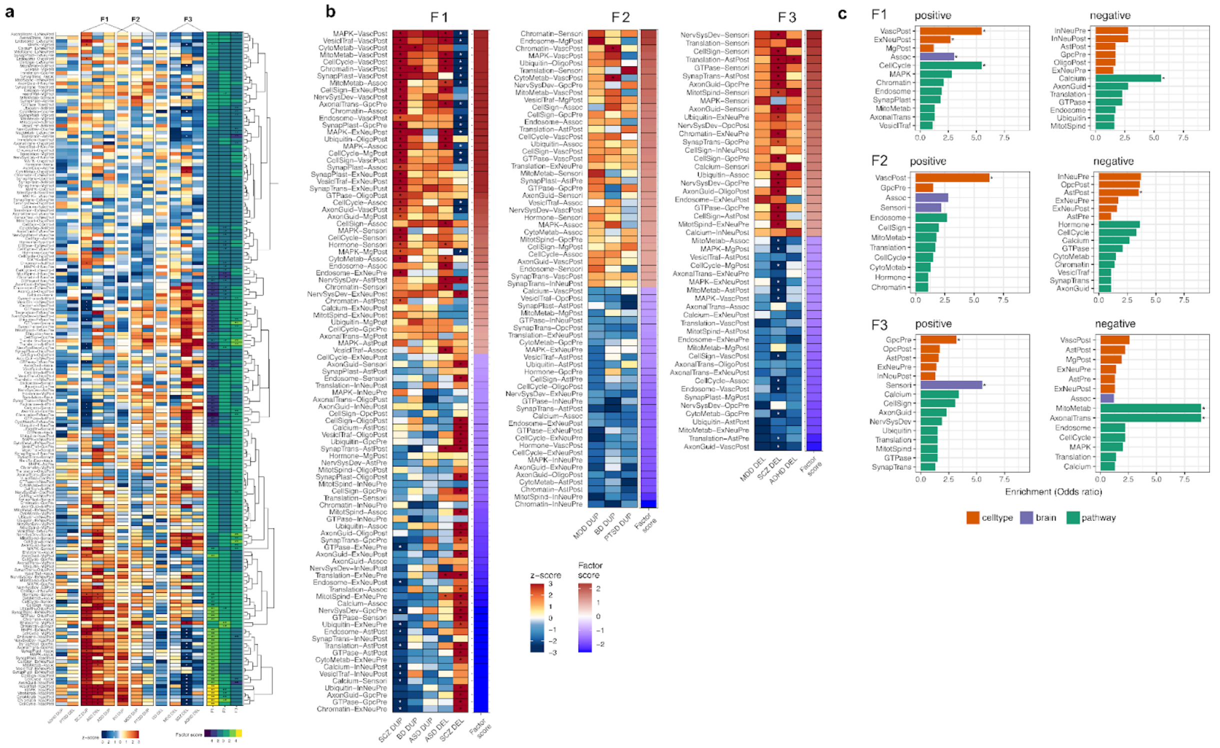

Fig. S6| Gene sets and functional terms linked to latent factors F1, F2 and F3 highlight neural processes that underlie orthogonal dimensions of gene-trait relationships. (**a**) a heatmap showing full gene set associations of all two-way pathway-stratified gene-sets (i.e., pathway-cell-type, and pathway-brain stratification). Red-white-blue color scale indicates gene set effect size from sample size weighted meta-analysis (z-score). Yellow-green-blue color scale indicates the F1, F2 and F3 factor scores for each gene set. Asterisks indicate gene set association that meets FDR correction in the combined summary statistics on 6 diagnostic categories (FDR<10%). **factor scores with absolute value >1. (**b**) To illustrate pathway-cell type and pathway-brain associations that contribute to factors, subsets of diagnosis-dosage and gene-sets were selected based on factor loadings and factor scores** for F1, F2 and F3 and sorted by factor score. (**c**) A bar plot highlighting pathway and cell-type terms that were enriched among positively or negatively loaded gene sets in panel B relative to the full summary statistics (fisher exact test P < 0.05).

medRxiv preprint doi: https://doi.org/10.1101/2025.07.11.25331381; this version posted July 16, 2025. The copyright holder for this preprint (which was not certified by peer review) is the author/funder, who has granted medRxiv a license to display the preprint in perpetuity.
It is made available under a CC-BY 4.0 International license .

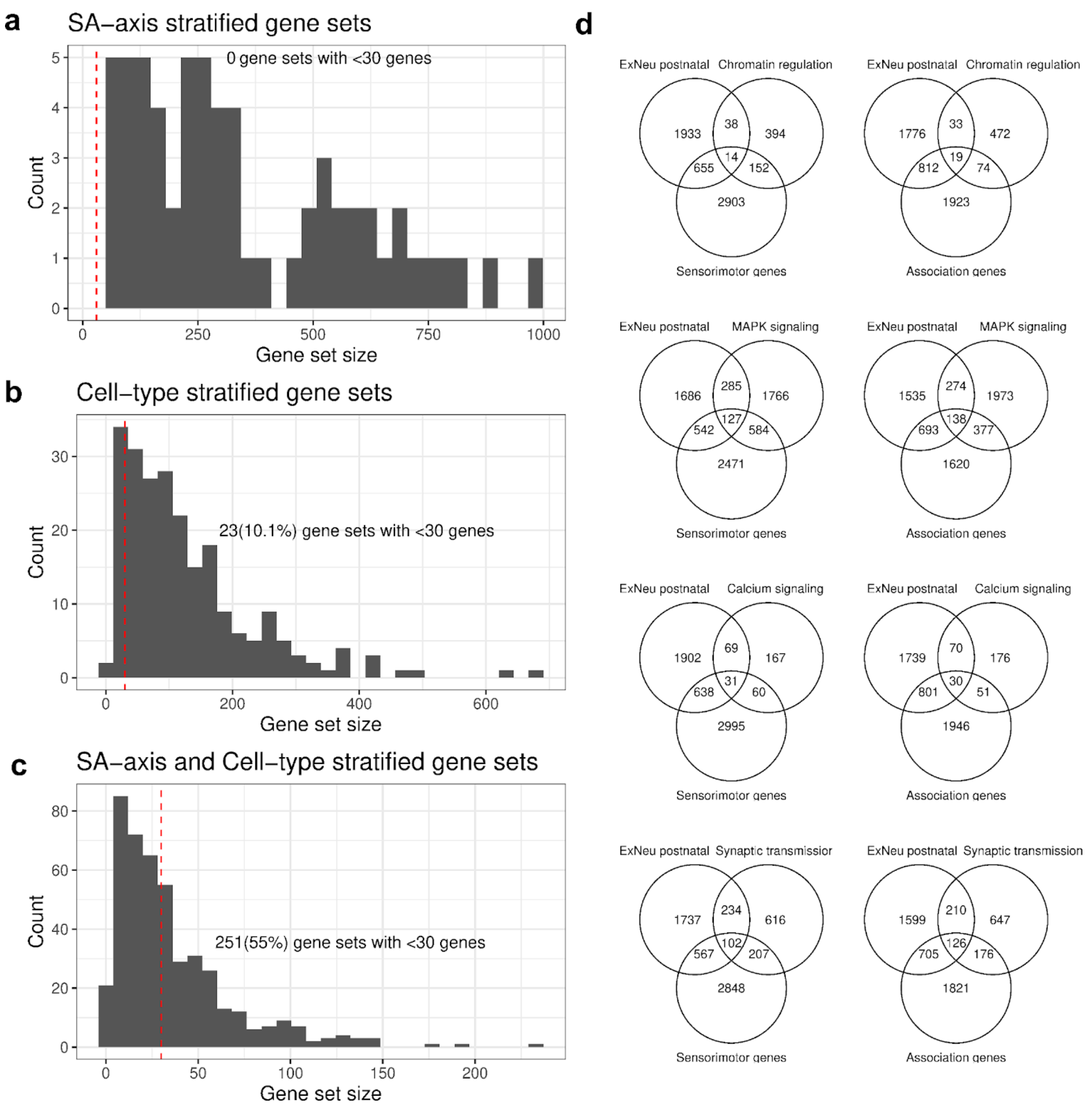

Fig. S7| Gene set size of stratified pathways. (**a**)-(**c**) Histograms display the distribution of gene set size when stratified the pathway clusters by (**a**) S-A axis, (**b**) 12 cell types, and (**c**) both S-A axis and cell types. Vertical dashed line indicates our 50 genes cut-off for gene sets to be included in the analysis. (**d**) Venn diagrams show the number of genes intersected between the major pathway gene sets (Chromatin regulation, MAPK signaling, Calcium signaling, and Synaptic transmission), Postnatal Excitatory Neurons, and Sensorimotor or Association genes.

medRxiv preprint doi: https://doi.org/10.1101/2025.07.11.25331381; this version posted July 16, 2025. The copyright holder for this preprint (which was not certified by peer review) is the author/funder, who has granted medRxiv a license to display the preprint in perpetuity.
It is made available under a CC-BY 4.0 International license .

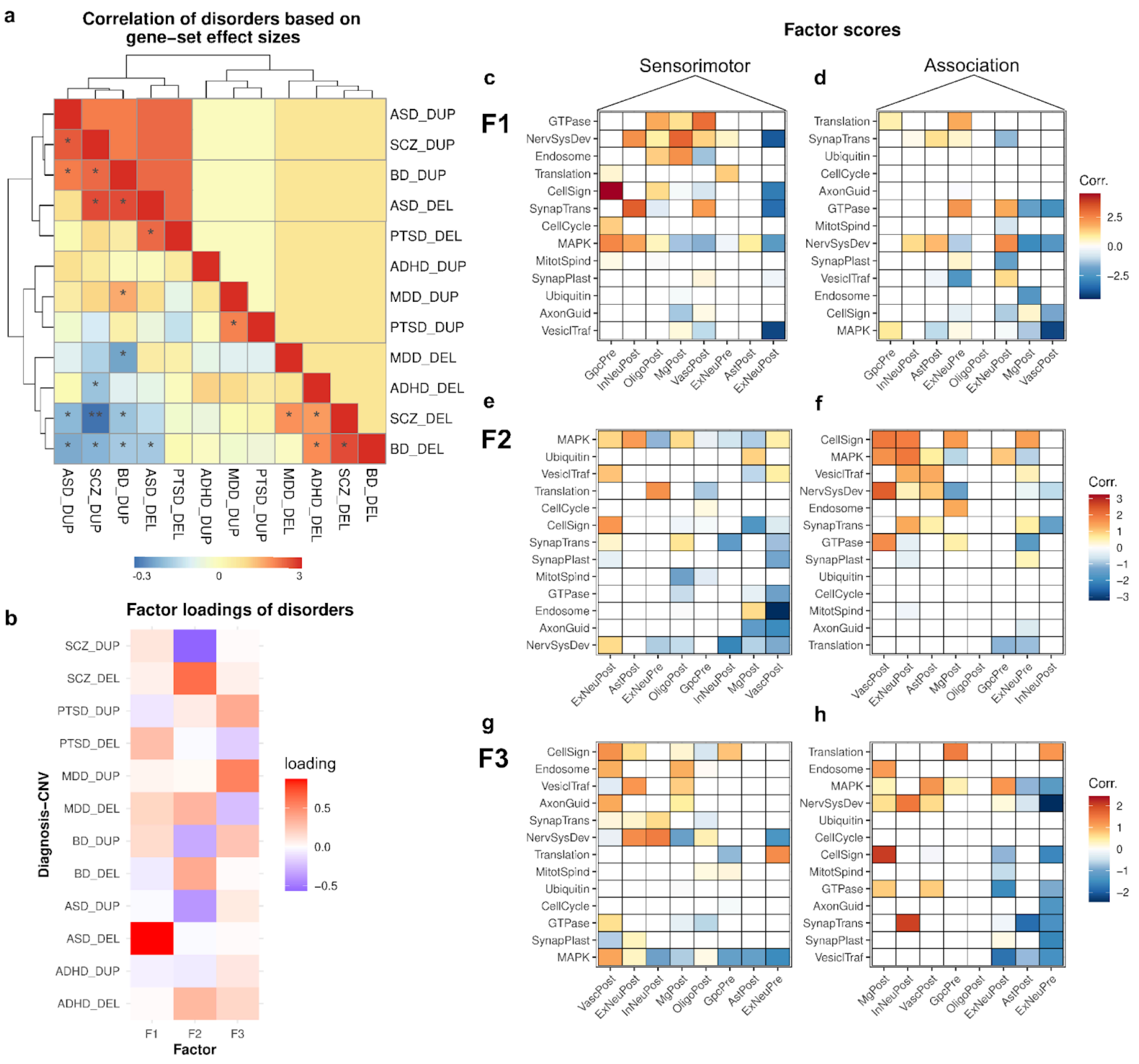

Fig. S8| Factor analysis of three-way pathway-celltype-brain stratification. The result shows that factor F2 and F3 are corresponding to the factor F1 and factor F2 of the main factor analysis result (**Fig 5.**) (**a**) Genetic correlation between diagnosis-dosage components. (**b**) Factor loadings. Factor scores for gene sets were shown as heatmaps for each of the three factors; where (**c**) and (**d**) correspond to factor F1, sensorimotor, and association gene sets, respectively. Similarly, (**e,f,g,h**) heatmaps show the factor scores for the factor F2, and F3.
